# UHPLC-MS/MS Assay for Quantification of Legubicin, a Novel Doxorubicin-Based Legumain-Activated Prodrug, and Its Application to Pharmacokinetic and Tissue Distribution Studies

**DOI:** 10.3390/molecules29040775

**Published:** 2024-02-08

**Authors:** Liyuan Ma, Qiaoling Yu, Meng Zhuang, Chen Yang, Yuan Liu, Yuling Li, Cheng Liu, Xiaoyan Shen, Yan Chang

**Affiliations:** 1 Department of Pharmacology, School of Pharmacy, Fudan University, Shanghai 201203, China; lyma@innostar.cn; 2 Shanghai Innostar Bio-Tech Co., Ltd., China State Institute of Pharmaceutical Industry, Shanghai 201203, China; qlyu@innostar.cn (Q.Y.); mzhuang@innostar.cn (M.Z.); cyang@innostar.cn (C.Y.); 3 Shanghai Affinity Bio-Pharmaceuticals Co., Ltd., Shanghai 201203, China; liuyuan@affinity.net.cn (Y.L.); liyuling@affinity.net.cn (Y.L.); chengliu@affinity.net.cn (C.L.)

**Keywords:** doxorubicin, Leu-DOX, legumain, prodrug, UHPLC-MS/MS, pharmacokinetics, tissue distribution

## Abstract

Legubicin, a novel prodrug based on doxorubicin, has both albumin-binding and legumain-activating properties. The aim of this study was to develop and validate a UHPLC-MS/MS method for investigating the in vivo pharmacokinetics and tissue distribution profiles of legubicin in rats and tumor-bearing mice following intravenous administration, and to compare this prodrug with the positive control drug doxorubicin. The study employed a UHLC-MS/MS method to determine the levels of albumin-bound of legubicin and two metabolites (free Leu-DOX and DOX) in plasma, tumor, and tissue samples. This method was validated for good selectivity, high sensitivity, excellent extraction recovery, and short run time. The results showed that legubicin was present in the circulation in vivo mainly in a protein-bound form with larger AUC values and lower clearance and distribution, and essentially released small amounts of doxorubicin. Compared to administration of equimolar doses of doxorubicin, legubicin showed increased exposure of the active drug in the tumor and decreased the level of the active drug in the heart and kidney. This study provides valuable information on the pharmacokinetics and tissue distribution of legubicin, implicating its potential as a novel and effective drug candidate for anti-cancer therapies.

## 1. Introduction

Doxorubicin (DOX), an anthracycline glycoside antibiotic, is a potent and versatile anti-tumor agent that is commonly employed, either as a monotherapy or in combination with other anti-neoplastic drugs, for the treatment of hematological (such as lymphoblastic and myeloid leukemia) and solid malignancies (such as soft tissue sarcoma, gastric cancer, recurrent glioma, breast cancer, etc.) [1]. Despite its efficacy, the clinical application of doxorubicin as an anti-neoplastic drug is impeded by its levels of certain systemic adverse effects such as damage to healthy tissues given the low specificity towards malignant cells, and its undesirable toxicity, particularly its dose-dependent cardiotoxicity and myelosuppression [2]. To overcome these restrictions, and improve the anti-tumor efficacy of doxorubicin, a prodrug of DOX, legubicin, was designed.

**Figure 1:**
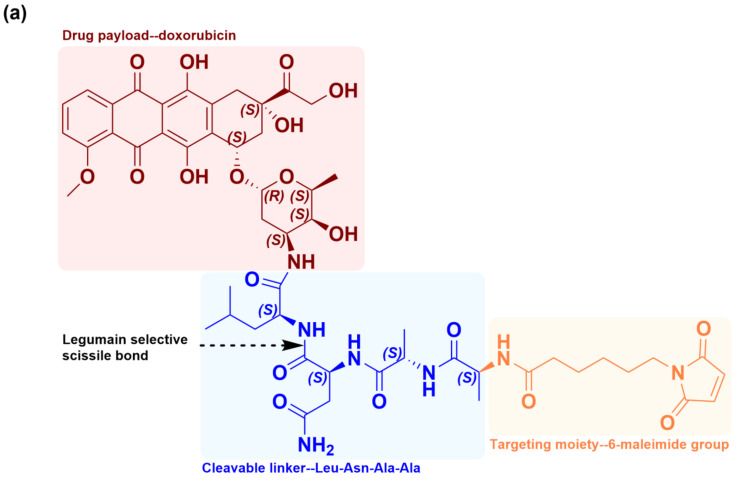

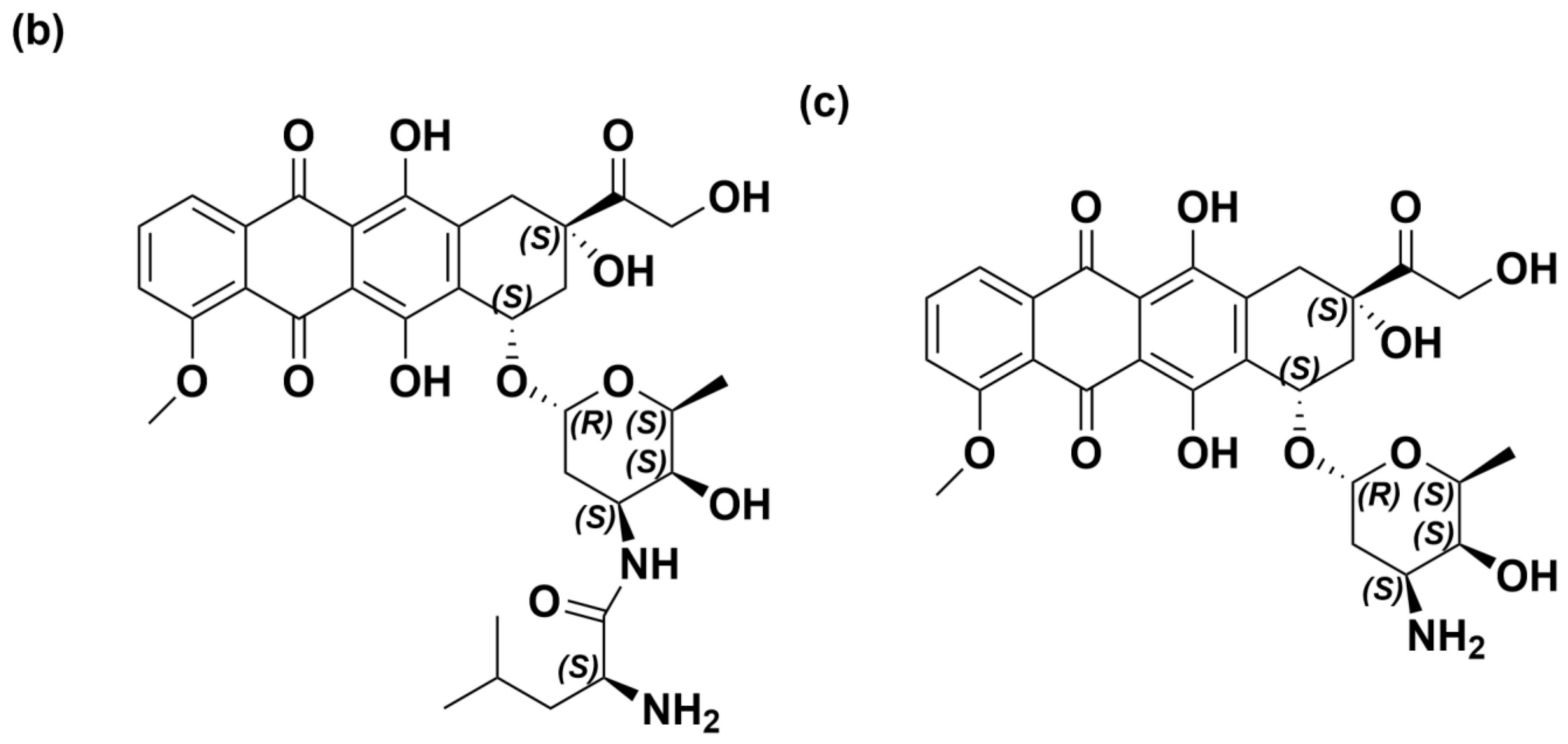
Chemical structures of Legubicin (**a**), Leu-DOX (**b**), and DOX (**c**).

The chemical structure of legubicin, as depicted in Figure 1a, comprises three components. It includes a tetrapeptide chain (Leu-Asn-Ala-Ala) attached to doxorubicin, rendering it inactive and transforming it into a non-toxic prodrug. Additionally, the peptide chain is capped with a 6-maleimide group at its terminus. The mechanism of action of legubicin involves the cleavage of asparaginyl bonds between leucine and asparagine by the enzyme legumain. This enzyme is overexpressed in cancerous tissues, leading to the release of the doxorubicin precursor N-L-leucyl-doxorubicin (Leu-DOX) under slightly acidic conditions, as illustrated in Figure 1b, and it does not exhibit enzymatic activity in neutral blood [3,4,5,6]. Following this enzymatic cleavage, Leu-DOX undergoes conversion to doxorubicin (DOX) (Figure 1c) through the action of tissue peptidases, such as cathepsins, which are widely expressed at tumor sites [5,7]. The presence of a 6-maleimide group (EMC) enables legubicin to covalently bind to the 34th cysteine residue of albumin in vivo, resulting in the formation of a macromolecular complex that hinders cellular penetration. Consequently, doxorubicin remains unable to enter the cell until it is activated by legumain in the tumor microenvironment. The utilization of plasma albumin as a pharmaceutical transporter is expected to exhibit a propensity for selective distribution towards tumor tissues as opposed to normal tissues, owing to the augmented permeability and retention of tumors caused by vascular leakage and compromised lymphatic drainage. Furthermore, it has the potential to reduce drug clearance and prolong the duration of action of small-molecule drugs [8].

In unpublished preclinical studies conducted at our institution, legubicin has demonstrated superiority over doxorubicin in preclinical investigations, exhibiting reduced toxicity in both rat and dog studies. The maximum tolerated dose (MTD) in acute toxicological assessments involving dogs was determined to be 15 mg/kg, representing a tenfold increase compared to doxorubicin. This MTD was twenty times higher than the effective dose, resulting in a significant reduction in the toxicity associated with doxorubicin. Furthermore, in an in vivo pharmacodynamic study utilizing a tumor immune model, legubicin exhibited immunotherapeutic properties by enhancing the activity of PD-1 antibodies, inducing immunological memory, and promoting cytotoxic T lymphocyte-mediated tumor cell destruction in a cured mouse.

The entry of legubicin into the circulation as an albumin conjugate and its specific activation by legumain in the tumor microenvironment may significantly modify its pharmacokinetic and tissue distribution behavior in vivo, compared to doxorubicin. Profiling the pharmacokinetics and tissue distribution of legubicin is essential for comprehending its efficacy and toxicity mechanisms. Moreover, the utilization of robust bioanalytical tools is particularly imperative for conducting the aforementioned investigations.

Currently, there is a lack of documented techniques for quantifying the albumin conjugate of legubicin in biological samples. This research aims to bridge this gap by developing and validating a rapid, sensitive and specific UHPLC-MS/MS method. This approach facilitates the concurrent assessment of the albumin conjugate of legubicin and its unbound metabolites in plasma, tumor, and tissues. Additionally, it is well-suited for investigating the pharmacokinetics and tissue distribution of these compounds subsequent to intravenous administration. Considering the novelty of legubicin as a prodrug of doxorubicin, a comparative analysis was also performed to assess the variances in pharmacokinetic and tissue distribution characteristics between the two compounds.

## 2. Methodologies, Validation, Results, and Discussion

### 2.1. Method Development

#### 2.1.1. Sample Preparation Method for Detecting Free Leu-DOX and DOX

The current methodology commonly employed in the literature for the detection of Leu-DOX and DOX involves the utilization of fluorescence detection after solid-phase extraction (SPE) pretreatment [5,9]. Nevertheless, this approach has been identified as labor-intensive, costly, and potentially deficient in the desired levels of specificity and sensitivity. In the present era, mass spectrometry has become the preferred method for detecting small-molecule drugs. As a result, we have devised an extraction technique that employs protein precipitation, and a liquid phase system which is compatible with mass spectrometry. This approach provides advantages in terms of its simplicity, rapidity, enhanced sensitivity, and cost-effectiveness.

Various published extraction methods, including protein precipitation using methanol [10,11,12], acetonitrile precipitation combined with sonication [13], liquid–liquid extraction [14,15,16], solid-phase extraction [17,18,19], and electromembrane extraction [20] have been employed for the extraction of Leu-DOX and its metabolized products or DOX and its metabolites from diverse matrices such as plasma, urine, and tissues. In this specific study, we examined the viability of a direct protein precipitation technique for the concurrent identification of Leu-DOX and DOX. This method demonstrated its practicality and cost-effectiveness, attaining sensitivities akin to those achieved through liquid–liquid extraction and solid extraction.

To ensure sufficient sensitivity, we added five times the volume of precipitant to precipitate rat plasma, and then centrifuged to separate the supernatant. Initially, we noted that different types of precipitant were reported in the literature to be feasible, such as methanol [12] and acetonitrile [13], and these were examined. It was observed that both methanol and acetonitrile brought about a matrix effect that enhanced the mass spectrometer response of Leu-DOX. When 20 µL of rat plasma was pre-treated with methanol or acetonitrile (100 µL), the matrix-effect values of Leu-DOX ranged from 114% to 121% at low, medium, and high concentration levels. To eliminate the matrix-enhancement effect, 0.1% formic acid and 5 mM ammonium formate were dissolved in methanol or acetonitrile. Furthermore, protein precipitation using methanol (0.1% formic acid + 5 mM ammonium formate) provided higher extraction efficiency and a better match to the initial mobile phase.

#### 2.1.2. Sample Preparation Method for Detecting Albumin Conjugate of Legubicin

**Figure 2:**
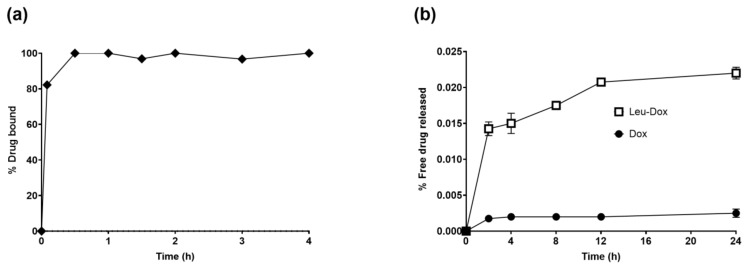
(**a**) Incubation kinetics of legubicin with HSA at room temperature (*n* = 1); (**b**) legubicin’s plasma stability profile (*n* = 4).

To determine the underlying substances of the assay, specifically the manner in which legubicin enters the bloodstream, whether as an unbound drug or in conjunction with albumin, legubicin was incubated with human serum albumin (HSA) at room temperature, and the resulting samples were analyzed using a fluorescence detector (excitation wavelength 470/emission wavelength 600). In a brief incubation period of five minutes, a significant proportion of the prodrug legubicin was observed to form a bond with albumin, indicating the rapid onset of the Michael addition-reaction (Figure 2a). To ascertain the stability of the linker within the legubicin structure and determine whether it would undergo breakage, resulting in the formation of Leu-DOX or DOX, the experiment on the plasma stability of legubicin was conducted. This investigation was crucial in preventing any interference with the detection of the free metabolites of legubicin (Leu-DOX and DOX). The stability of the albumin conjugate of legubicin in rat plasma was demonstrated by the release of no more than 0.023% and 0.003% of free Leu-DOX and DOX, respectively, over a 24 h period (Figure 2b). Additionally, it was demonstrated that the inclusion of the albumin conjugate of legubicin did not exert any discernible influence on the detection of unbound metabolites, specifically, Leu-DOX and DOX.

Nevertheless, the detection of albumin-binding drugs through mass spectrometry is hindered due to their considerable molecular size. To address this issue, we have developed a novel technique that entails enzymatically transforming these drugs into smaller molecules suitable for mass-spectrometry analysis. Subsequently, the converted molecules undergo the same pretreatment procedure as free Leu-DOX and DOX. Legumain exhibited restricted substrate specificity and cleaved the asparagine C-terminal peptide, resulting in doxorubicin linked to leucine (Leu-DOX). In the meantime, Leu-DOX was one of the metabolites inherently found in the blood. Therefore, the legubicin–albumin conjugate concentration was determined according to the following steps: step one performed an in vitro legumain digestion assay to detect the concentration of Leu-Dox after cleavage; step two measured the free Leu-Dox concentration, which corresponded to the native concentration in the blood; step three subtracted the concentrations from steps one and two.

**Figure 3:**
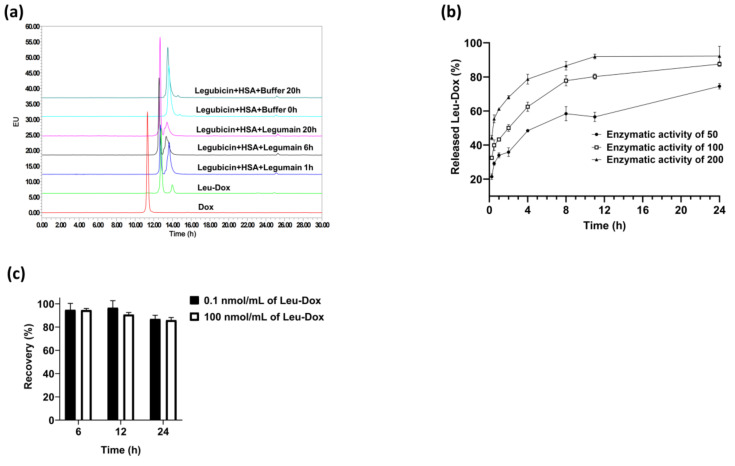
(**a**) Chromatograms of Dox (red); Leu-Dox (green); legubicin–HSA conjugate (powder blue and ink blue); and legubicin–HSA conjugate incubated with legumain for 1 h (blue), 6 h (black), and 20 h (pink). The specific liquid-phase conditions are shown as follows. Column: Agilent Eclipse Plus 4.6 × 250 mm, 5 µm; mobile phase A: 0.1% trifluoroacetic acid in water; mobile phase B: acetonitrile. Flow rate: 1.0 mL/min; HPLC: Waters 2695; detector: Waters 2475 detector (ex470/em600); column temperature: 30 °C; injection volume: 10 µL; run time: 30 min; needle wash solvent: 50% acetonitrile in water; gradient elution: starting 20% B, increasing from 20% to 45% B in 15 min, increasing from 45% to 80% B in 15 to 20 min, maintained for 5 min, then decreasing to 20% B in 0.1 min, and continuing until the end of 30 min. (**b**) Kinetics of Leu-Dox release from legubicin (black) and legubicin–albumin conjugate (blue) in the presence of legumain (*n* = 4). (**c**) Stability of Leu-Dox in this enzymatic system (*n* = 3).

To observe the role of legumain, HSA-Legubicin was co-incubated with legumain, and the subsequent release of Leu-DOX was quantified using high-performance liquid chromatography. The HSA-Legubicin peak experienced a decline lasting over one hour, followed by the emergence of a new peak at the retention time of 12.78 min, resembling the Leu-DOX standard. These findings indicate that legumain facilitates the liberation of Leu-DOX. With the extension of the incubation period, the Leu-Dox peak gradually intensified, while the HSA-Legubicin peak progressively diminished (Figure 3a).

The enzymatic-hydrolysis efficiency of legubicin was determined by incorporating legumain and varying the enzyme activity while monitoring the legubicin content at different enzymatic-hydrolysis time points. This comparison aimed to assess the impacts of different enzymatic-hydrolysis conditions on the efficiency of the process. Furthermore, it should be noted that the presence of mucin in the enzyme reagent may lead to emulsification or column blockage. Following a 24 h period of HSA-Legubicin interaction with the activating enzyme, it was observed that Leu-DOX was fully liberated in groups exhibiting enzymatic activities of 100 and 200 (Figure 3b). The optimal conditions for enzymatic hydrolysis, as described in Section 3.7.1, have thereby been finalized.

These findings indicate that the prodrug legubicin forms a covalent bond with HSA while concurrently releasing the unbound drug upon activation by legumain.

The assessment of Leu-DOX stability in the aforementioned enzymatic systems warrants careful consideration. Consequently, plasma samples containing Leu-DOX were intentionally supplemented to assess the potential impacts of these methods on the precise determination of the enzymatic digestion of the legubicin plasma protein complex, which is utilized for determining the total Leu-DOX post-digestion. This preliminary inquiry revealed that Leu-DOX exhibited steadfast stability and remained unaltered throughout the enzymatic digestion procedure (Figure 3c). This observation validated the accuracy and reliability of determining the concentration of the albumin conjugate of legubicin by subtracting the background free Leu-DOX concentration from the Leu-DOX concentration after enzymatic cleavage.

### 2.2. Method Validation

#### 2.2.1. Selectivity, Specificity, and Carry-Over

As presented in Figure S1, no observable interference peak was present in the six blank plasma or homogenate samples at the peak regions for each analyte or phenacetin (IS), and no interference between analyte and IS was observed, thus indicating high selectivity and specificity for the proposed method. The results of carry-over showed that the former injection had no effect on the quantitation of the latter.

#### 2.2.2. Linearity

The calibration ranges, calibration curves, correlation coefficients, limit of detection (LOD), and limit of quantification (LOQ) of the analytes in each biological sample are included in Table 1. All of the correlation coefficients (r) were above 0.99, indicating good fitness of the calibration curves for the analytes in various biological matrices.

#### 2.2.3. Accuracy and Precision

The results of intra-batch and inter-batch accuracy and precision analyses of each of the analyte QCs at the four concentration levels in rat plasma and mouse biological matrices are presented in Table 2 and Table S1, respectively. The bias from the theoretical value was within 15% on both an intra-batch and inter-batch basis. In addition, values for intra-batch and inter-batch precision were less than 15%. These data showed that the established methodology was robust and reproducible.

#### 2.2.4. Extraction Recovery and Matrix Effect

The determined results for extraction recovery and matrix effect in rat plasma and mouse biomatrices are summarized, respectively, in Table 3 and Table S2. The recoveries varied from 89.7% to 102.1%, with RSD values ranged from 3.3 to 9.9%, for Leu-DOX, DOX, and albumin conjugate of legubicin in rat plasma. The matrix-effect values were within the range of 84.3–108.8%, with RSDs of 2.8–10.9%. The extraction process of rat plasma was demonstrated to be consistent and repeatable, as well as free from apparent matrix interference.

#### 2.2.5. Dilution Integrity

The inaccuracy and imprecision values of the dilution integrity of the analytes after a 10-fold or 20-fold dilution were within 15% (as listed in Table S3).

#### 2.2.6. Stability

Table 4 and Table S4 summarize all investigated stability data for the analytes in rat and mouse bio-samples, respectively, indicating that Leu-DOX, DOX, and albumin conjugate of legubicin exhibited no appreciable degradation under the previously mentioned conditions. The specific stability periods of the analytes in the individual biological matrices have been detailed in the tables above.

### 2.3. Pharmacokinetic Study in SD Rats

#### 2.3.1. Single-Dose Studies

**Figure 4:**
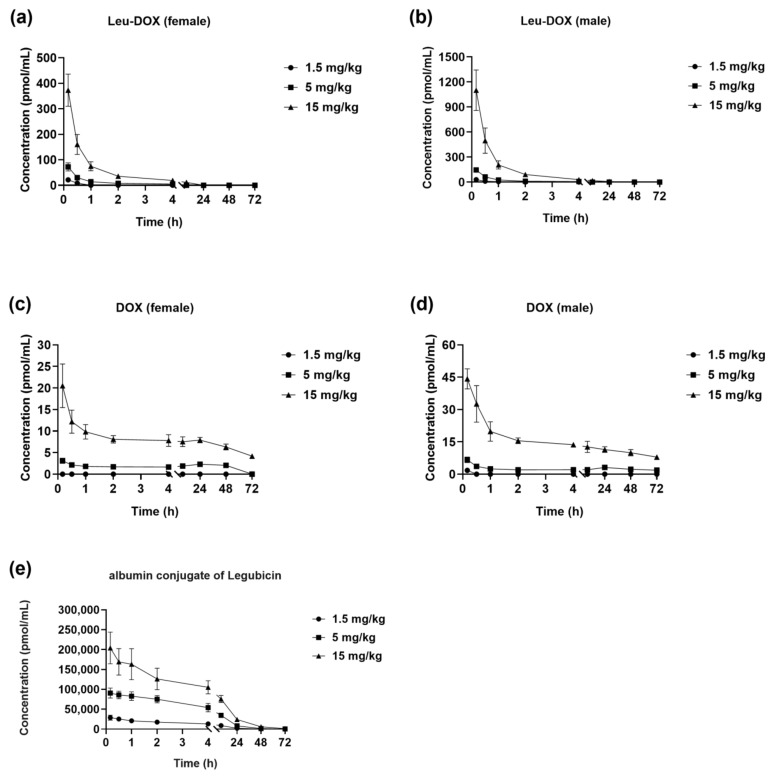
Profiles of mean plasma concentration versus time of (**a**) Leu-DOX (female), (**b**) Leu-DOX (male), (**c**) DOX (female), (**d**) DOX (male), and (**e**) albumin conjugate of legubicin, after single dose administration (1.5, 5, and 15 mg/kg, respectively) in Sprague-Dawley rats (**a**–**d**, *n* = 3; **e**, *n* = 6).

The validated methodology was employed to ascertain the presence of Leu-DOX, DOX, and albumin conjugate of legubicin in plasma samples obtained from a pharmacokinetic study of legubicin in four-cycle SD rats. The rats were administered legubicin intravenously at doses of 1.36, 4.52, and 13.6 µmol/kg doxorubicin equivalents in order to conduct single-dose studies and determine the concentrations of Leu-DOX, DOX, and albumin conjugate of legubicin at various time intervals. The average plasma concentration–time profiles of Leu-DOX, DOX, and albumin conjugate of legubicin are depicted in Figure 4. The concentrations of Leu-DOX, DOX, and the albumin conjugate of legubicin exhibited an upward trend with escalating doses. Notably, the exposure levels of the albumin conjugate of legubicin, as measured by C_max_ and AUC_last_, were significantly higher, by two to five orders of magnitude, compared to those of Leu-DOX and DOX. This observation implies that legubicin predominantly circulates in the bloodstream in a protein-bound state following administration, with minimal to no release of DOX. Consequently, it can be inferred that legubicin may offer a heightened safety profile in comparison to DOX in vivo.

The pharmacokinetic parameters are outlined in Table 5. As DOX was detected at only a few time points in the low-dose single-dose group (group 1), relevant pharmacokinetic parameters were not calculated. The maximum plasma concentrations (C_max_) of legubicin–albumin conjugate were achieved rapidly following intravenous administration, with median time to C_max_ (T_max_) values within 0.167–1 h for all dose-groups. The clearance (Cl_obs_) values of the three doses were 6.4 ± 1.53 mL/h/kg, 5.33 ± 1.05 mL/h/kg, and 6.87 ± 1.16 mL/h/kg, respectively. The half-life (t_1/2z_) values were 8.43 ± 2.6 h, 9.72 ± 1.01 h, and 10.7 ± 2.31 h, respectively. The AUC_last_ values were 218,000 ± 47,400 h*pmol/mL, 869,000 ± 152,000 h*pmol/mL, and 2,000,000 ± 324,000 h*pmol/mL, respectively. The AUC_INF_obs_ were estimated to be 222,000 ± 47,600 h*pmol/mL, 873,000 ± 155,000 h*pmol/mL, and 2,020,000 ± 331,000 h*pmol/mL, respectively. The C_max_ values of the three doses were 28,900 ± 6310 pmol/mL, 97,300 ± 8270 pmol/mL, and 204,000 ± 39,800 pmol/mL, respectively. As the dosage of legubicin at a ratio of 1:3.3:10 was increased, the C_max_, AUC_last_, and AUC_INF_obs_ values rose, at ratios of 1:3:7, 1:4:9, and 1:4:9, respectively. A power-function model was used to fit the relationship between exposure level (C_max_, AUC_last_, and AUC_INF_obs_) parameters and dose, and the power exponent (parameter β) and its 90% confidence interval were obtained (see Table S5). The increase of C_max_ was in a slightly less than dose-proportional manner throughout the intravenous dose range, and the AUC values increased in proportion to the dose.

#### 2.3.2. Multiple-Dose Studies

**Figure 5:**
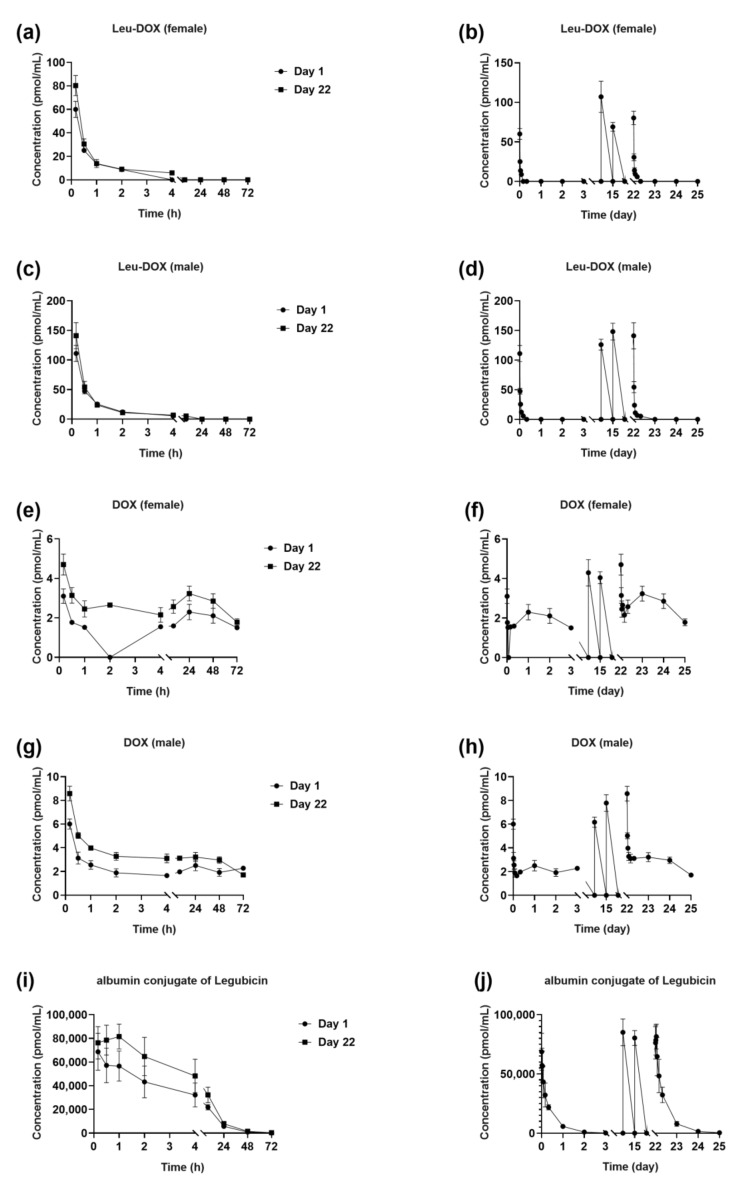
Profiles of mean plasma concentration versus time of (**a**,**b**) Leu-DOX (Female), (**c**,**d**) Leu-DOX (Male), (**e**,**f**) DOX (Female), (**g**,**h**) DOX (Male), and (**i**,**j**) albumin conjugate of legubicin. At left, the comparison between days 1 and 22, and at right, the continuous weekly dosing of 1.5 mg/kg of legubicin for four weeks in Sprague-Dawley rats ((**a**–**h**), *n* = 3; (**i**,**j**), *n* = 6).

The mean plasma concentration versus time profiles of Leu-DOX, DOX, and albumin conjugate of legubicin in the plasma of SD rats are depicted in Figure 5, following 4 consecutive weeks of weekly intravenous injections of legubicin at 5 mg/kg.

The initial administration of legubicin on day 1 resulted in the albumin conjugate of legubicin’s observed values of 560,000 ± 90,900 h*pmol/mL, 563,000 ± 91,200 h*pmol/mL, and 68,700 ± 15,600 pmol/mL for AUC_last_, AUC_INF_obs_, and C_max_, respectively. Similarly, the final injection on day 22 yielded AUC_last_, AUC_INF_obs_, and C_max_ values of 808,000 ± 156,000 h*pmol/mL, 814,000 ± 156,000 h*pmol/mL, and 88,600 ± 12,200 pmol/mL, respectively.

The study’s findings indicate that the accumulation ratios (AR) of the AUC_last_, AUC_INF_obs_, and C_max_ for the albumin conjugate of legubicin were observed to be approximately 1.4, 1.4, and 1.3, respectively, when comparing the ratios of AUC_last_, AUC_INF_obs_, and C_max_ on day 22 to those on day 1. Table 6 demonstrates that there was no significant accumulation of the albumin conjugate of legubicin, Leu-DOX, or DOX with repeated administration of legubicin.

The main multiple-dose pharmacokinetic parameters of legubicin were compared to published values for doxorubicin and DOXO-EMCH (i.e., doxorubicin (6-maleimidocaproyl) hydrazone). There were significant differences observed between legubicin and doxorubicin in terms of the area under the curve (AUC), maximum plasma concentration (C_max_), clearance (CL), and volume of distribution (V_z_), with variations ranging from two to three orders of magnitude. The superior AUC value of legubicin compared to doxorubicin demonstrates the benefits of a tumor-microenvironment-activated prodrug, as it exhibits prolonged plasma persistence, thereby enabling extended tumor exposure and enhanced targeted therapeutic effects. According to the literature, the AUC values for doxorubicin and DOXO-EMCH are 1.4 h µM and 536 h µM, respectively, when administered at doses equivalent to 2.5 mg/kg of doxorubicin [21]. The clearance (CL) value of legubicin was approximately 5.75 mL/h/kg, significantly lower than that of doxorubicin (approximately 2553 mL/h/kg), and marginally lower than DOXO-EMCH (approximately 7.9 mL/h/kg) [21]. The V_z_ value of legubicin was determined to be 0.082 L/kg, whereas doxorubicin exhibited a V_z_ value of 72 L/kg, as reported in the literature [21]. There was a lack of statistically significant disparity observed between the half-life of legubicin and the terminal half-life of doxorubicin [21]. The pharmacokinetic properties of legubicin demonstrate a low clearance and small distribution volume, implying its potential for prolonged efficacy, localized diffusion, and targeted therapeutic applications.

### 2.4. Tissue-Distribution Study in Tumor-Bearing Mice

**Figure 6:**
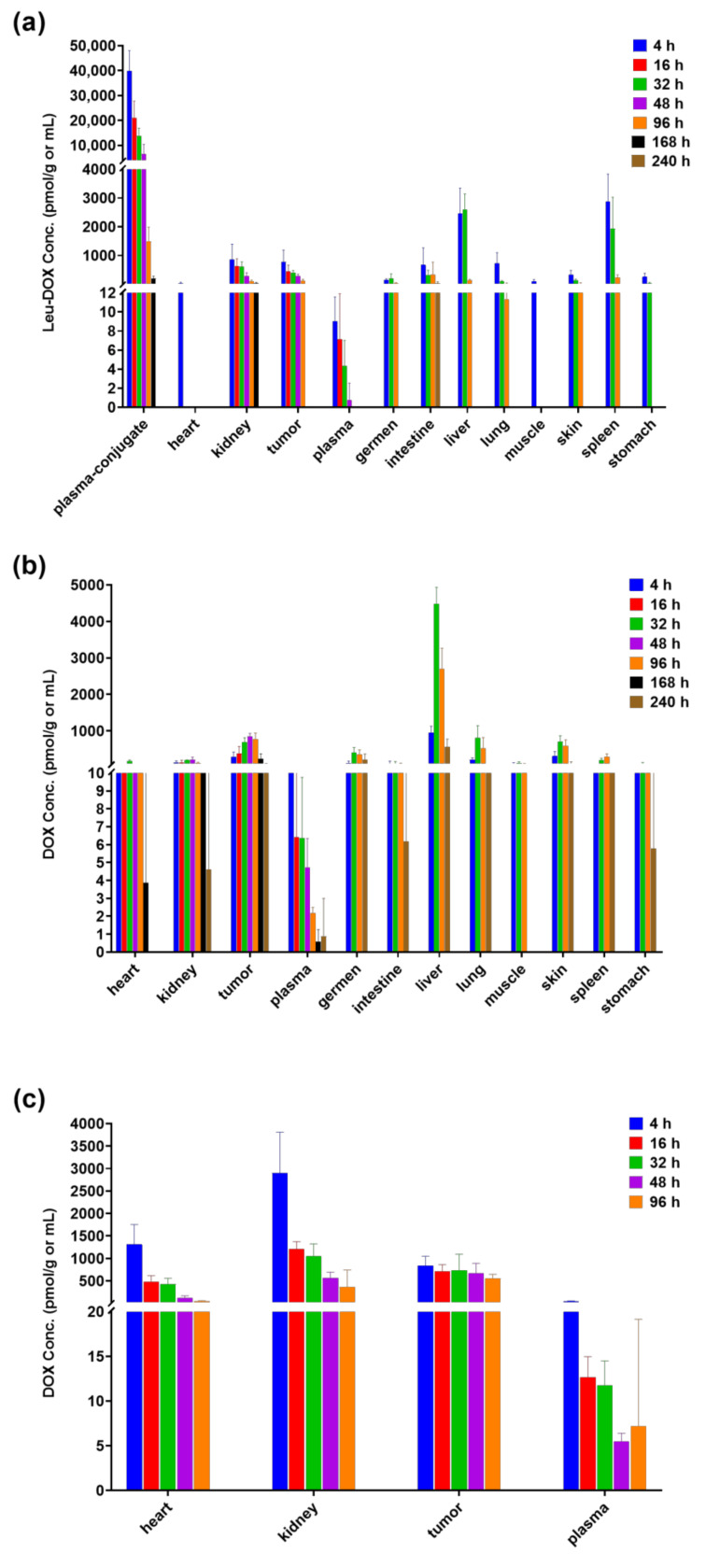
Mean tissue–drug/plasma–drug concentrations in tissues and plasma of tumor-bearing mice after intravenous administration of legubicin of (**a**) Leu-DOX; (**b**) DOX, or administration of doxorubicin (**c**) (*n* = 6).

The concentrations of free Leu-Dox and Dox in various tissues at specified time points are depicted in Figure 6 subsequent to the administration of legubicin (9.05 µmol/kg) to mice with tumors. Following the administration of legubicin, neither the metabolite doxorubicin nor free Leu-Dox permeated the brain. The study revealed that the metabolite free-Leu-Dox exhibited its highest concentration in the liver, followed by the spleen, small intestine, kidney, tumor, lung, skin, gonads, stomach, plasma, skeletal muscle, and heart. Similarly, the distribution of the metabolite doxorubicin displayed a concentration trend, with the liver having the highest levels, followed by the tumor, skin, lung, gonads, spleen, kidney, stomach, small intestine, skeletal muscle, heart, and plasma. It is worth noting that the tumor exhibited significantly higher levels of doxorubicin compared to the other tissues, except for the liver.

The depiction of the distribution of doxorubicin in kidney, tumor, and heart tissues can be observed in Figure 6c. Following the administration of doxorubicin, a prompt in vivo distribution was observed, with all tissues reaching their maximum concentration (C_max_) at the initial sampling point (4 h). The order of exposure, in relation to Dox distribution, was determined to be kidney > tumor > heart > plasma.

**Figure 7:**
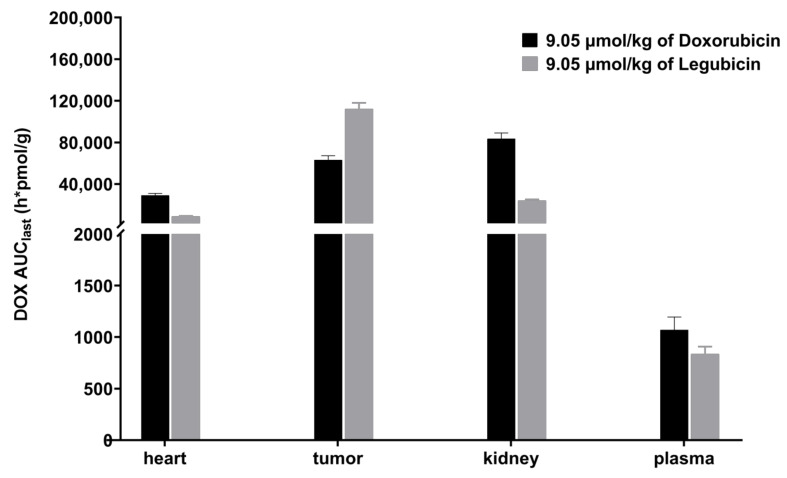
Comparison of doxorubicin exposure (AUC_last_) in plasma, heart, kidney, and tumor, after intravenous administration of doxorubicin or legubicin, in tumor-bearing mice (*n* = 6).

The distribution of doxorubicin in the heart, kidney, tumor, and plasma following intravenous administration of legubicin or doxorubicin in mice with tumors is summarized in Table 7. The tissue-to-plasma (T/P) concentration ratio exceeded 1, indicating that doxorubicin is localized there. Additionally, a comparison of doxorubicin exposure (AUC_last_) is illustrated in Figure 7. In comparison to the equimolar dose of doxorubicin administration, it was observed that legubicin exhibited a tendency to enhance doxorubicin exposure in tumor tissue while reducing its exposure in the heart and kidney. Specifically, the analysis revealed a 3.3-fold decrease in doxorubicin exposure (AUC_last_) in the heart, a 3.5-fold decrease in the kidney, and a 1.8-fold increase in tumor doxorubicin exposure (AUC_last_) subsequent to the administration of legubicin. As a result of diminished absorption and toxicity in non-malignant tissues such as the heart and kidney, a greater quantity of legubicin was accessible for therapeutic use, leading to elevated drug concentrations within intra-tumor cells, when compared to the administration of doxorubicin. In summary, the concentration of doxorubicin in tumor tissue exhibited a gradual-activation kinetic profile, gradually increasing from low to high concentrations following legubicin injection, whereas doxorubicin administration displayed a rapid-elimination kinetic process. This observation further implies a prolonged efficacy-duration of legubicin.

## 3. Materials and Methods

### 3.1. Chemicals and Reagents

Legubicin (purity: 97.95%), Leu-DOX (purity: 98.6%), Doxorubicin hydrochloride (Content: 99.0%), and Phenacetin (Internal standard, IS, purity: 100.0%) were obtained from the Sandia Medical Technology (Shanghai) Co., Ltd. (Shanghai, China); Shanghai Affinity Bio-pharmaceuticals Co., Ltd. (Shanghai, China); Zhejiang Hisun Pharmaceutical Co., Ltd. (Taizhou, China); and Sigma Aldrich Trading Co., Ltd. (Shanghai, China), respectively.

Ultrapure water was produced using the Millipore Milli-Q advantage water purification system (Merck, Albany, GA, USA). PBS was obtained from HyClone. Methanol and acetonitrile (HPLC grade) were procured from Merck (Darmstadt, Germany). Dimethyl sulfoxide (HPLC grade) was purchased from Sinopharm Chemical Reagent Co., Ltd. (Shanghai, China). Ammonium formate and ammonium acetate were acquired from Sigma Aldrich (Shanghai) Trading Co., Ltd. Formic acid (Chromatographic grade) was obtained from Shanghai Aladdin Bio-Chem Technology Co., Ltd., China. Blank Rat K2EDTA plasma was supplied by Shanghai InnoStar Bio-tech Co., Ltd. (Shanghai, China). Legumain and assay buffer were obtained from Shanghai Affinity Bio-pharmaceuticals Co., Ltd. The 0.9% NaCl solution for injection was provided by Qingzhou Yaowang Pharmaceutical Co., Ltd. (Weifang, China). Legumain enzyme and enzyme digestion buffer were from Shanghai Affinity Bio-pharmaceuticals Co., Ltd. (Shanghai, China).

### 3.2. Animals

The Sprague-Dawley rats were procured from Beijing Vital River Laboratory Animal Technology Co., Ltd. (Beijing, China). The HT1080 tumor-bearing mice were obtained from Shanghai Lingchang Biotechnology Co., Ltd. (Shanghai, China). All animals were housed, with separate cages for males and females, under standard experimental conditions (20–26 °C, relative humidity at 40–70%, under a 12 h light/dark cycle) and fasted for 12 h before the experiment, with free access to water.

### 3.3. Instrumentation and UHPLC-MS/MS Parameters

The UHPLC-MS/MS analysis was conducted with the use of an Acquity UPLC unit composed of a binary pump, degasser, sample manager and autosampler, and column oven, combined with a Turbo Ionspray^TM^ source built into the 5500 QTRAP (for analysis of rat plasma)/QUAD (for analysis of mouse plasma and tissues) mass spectrometer (AB Sciex, Concord, ON, Canada). An ACQUITY UPLCTM BEH C18 column (particle size: 1.7 µm, 2.1 mm × 50 mm, i.d., Waters Corporation, Milford, MA, USA) was utilized to achieve chromatographic separation at a column temperature of 40 °C.

A gradient elution was carried out for analysis of rat plasma samples using 0.1% formic acid aqueous solution (mobile phase A) and 0.1% formic acid in acetonitrile (mobile phase B). The gradient elution was run with the following parameters: Initially, B remained at 10% for 0.2 min, and then was raised linearly from 10% to 90% over the next 1.6 min, remained at 90% for 0.4 min, and then decreased from 90% to 10% over 0.1 min before being stabilized at 10% for 0.2 min. Compounds were eluted for 2.5 min at a flow rate of 0.6 mL/min from a volume of 5 µL (detection of free Leu-DOX and DOX in rat plasma)/3 µL (detection of total Leu-DOX in rat plasma after enzymatic hydrolysis by legumain) of the injection sample.

The chromatographic conditions used in the analyses of mouse plasma and tissues were similar.

Multiple-reaction monitoring (MRM) signals were recorded with the electrospray ionization positive mode (ESI+). Since isotope-labeled internal standards are not readily available and costly, phenacetin was chosen for its chromatographic retention behavior in close proximity to the analytes. The MRM transitions selected for each analyte and the detailed mass parameters for Leu-DOX, DOX, and phenacetin are outlined in Table 8. Product ion mass spectra of Leu-DOX, DOX, and IS are shown in Figure S2. The source/gas-related parameters were as follows: ion-spray voltage (IS), 5500 V; source temperature, 500 °C; curtain gas, nebulizer gas (GS1), and auxiliary gas (GS2) were set at 40, 60, and 60 psi, respectively; collision-activated dissociation gas (CAD) was set to Medium and 9 for rat and mouse bio-samples, respectively. The assay conditions for total Leu-DOX after legumain enzymatic hydrolysis are the same as those for free Leu-DOX and DOX.

### 3.4. Stock and Working Solutions Preparation

Legubicin, Leu-DOX, DOX, and phenacetin stock solutions were individually prepared in dimethyl sulfoxide (DMSO) at 1 mM and stored at −80 °C.

A collection of concentrations of Leu-DOX and DOX working solution mixtures were obtained by mixing stock solutions of Leu-DOX and DOX, and further diluting with methanol. Appropriate dilutions were made with methanol to obtain working solutions of legubicin at different concentrations. Phenacetin (IS) stock solution was diluted with methanol (0.1% formic acid + 5 mM ammonium formate) to yield 3 pmol/mL and 5 pmol/mL IS working solutions for pharmacokinetic study in rats. Additionally, 5 pmol/mL and 50 pmol/mL IS working solutions for mouse tissue distribution study were obtained with methanol (0.1% formic acid + 5 mM ammonium acetate) dilution. Prior to use, all solutions were freshly prepared.

### 3.5. Calibration Standards and Quality Control Samples Preparation

Calibration standards were prepared at the following concentrations: Leu-DOX (5, 25, 50, 250, 500, 2500, 5000 pmol/mL for rat plasma; 3, 6, 15, 30, 150, 300, 1500, 3000 for mouse plasma; 9, 15, 30, 150, 300, 1500, 3000, 9000 pmol/mL for tumor and tissues), DOX (1.5, 7.5, 15, 75, 150, 750, 1500 pmol/mL for rat plasma; 1, 2, 5, 10, 50, 100, 500, 1000 pmol/mL for mouse plasma; 3, 5, 10, 50, 100, 500, 1000, 3000 pmol/mL for tumor and tissues), and albumin conjugate of legubicin (0.1, 0.3, 1, 5, 10, 50, 100 nmol/mL for rat and mouse plasmas). The concentrations of the quality control (QC) samples were set at 4 levels (the lowest limit of quantitation (LLOQ), low, medium, and high concentrations) as follows: 5, 10, 200, and 4000 pmol/mL for Leu-DOX in rat plasma; 3, 9, 120, and 2400 pmol/mL for Leu-DOX in mouse plasma; 9, 27, 2400, and 7200 pmol/mL for Leu-DOX in tumor and tissues; 1.5, 3, 60, and 1200 pmol/mL for DOX in rat plasma; 1, 3, 40, and 800 pmol/mL for DOX in mouse plasma; 3, 9, 800, and 2400 pmol/mL for DOX in tumor and tissues; and 0.1, 0.2, 4, and 80 nmol/mL for albumin conjugate of legubicin in rat and mouse plasmas.

To verify the absence of interference, a zero sample (without analytes, but with IS) and a blank sample (no analyte, and no IS) were included in each analytical batch. The calibrators and QC samples were freshly made on the same day that sample analysis occured, and pre-treated by the same procedures as the study samples, as outlined in Section 3.7.1.

### 3.6. Preparation of Administration Formulation

Lyophilized legubicin (purity: 94.3%, 10 mg/vial) and special solvent for legubicin (propylene glycol: ethanol: normal saline = 40:10:50) were sourced from Shanghai Affinity Bio-pharmaceuticals Co., Ltd. (Shanghai, China). Legubicin was reconstituted by adding 3 mL of sterile special solvent to obtain the test article solution at 3.143 mg/mL, which was kept in darkness at room temperature before administration.

### 3.7. Sample Pretreatment

#### 3.7.1. Plasma Samples

For free Leu-DOX and DOX, an aliquot of 20 µL of rat plasma was transferred into a 1.5-mL Eppendorf (EP) tube and precipitated with 100 µL of IS working solution (3 pmol/mL). The mixture was vortexed for 3 min, and then centrifuged at 22,000× *g* at 4 °C for 10 min. A final analysis was conducted by injecting 5 µL supernatant into a UHPLC-MS/MS system. Mouse plasma samples were treated in the same way as the rat plasma samples, except for protein precipitation, with 200 µL of 5 pmol/mL IS working solution and injection of 2 µL of supernatant for analysis.

For total Leu-DOX after enzymatic hydrolysis by legumain, the following processes were used. (1) For the hydrolysis process, an aliquot of 25 µL of rat/mouse plasma was transferred into an Eppendorf tube, and then 50 µL of enzyme digestion buffer and 25 µL of legumain enzyme were placed into the tube. The mixture was vortexed for 1 min, and then incubated in a water bath for 24 h at 37 °C for enzymatic hydrolysis. (2) For the protein precipitation process, 1000 µL of IS working solution (5 pmol/mL and 50 pmol/mL for rat and mouse plasmas, respectively) was added to the enzymatic-hydrolysis solution. Then, the sample was vortexed for 3 min and centrifuged at 22,000× *g* for 10 min at 4 °C. Analyses were performed on the 3 µL aliquot of the solution used in the UHPLC-MS/MS system.

#### 3.7.2. Tumor and Tissue Samples

Tumor-bearing mouse tumor/individual tissue samples were homogenized by adding PBS at a ratio of 1:5 (e.g., 1 g of tumor sample to 5 mL of PBS) to make a tumor/tissue homogenate. A quantity of 60 µL of homogenate was taken as a tumor/tissue sample and processed in the same manner as the mouse plasma sample, with the exception of 240 µL of IS working solution used for protein precipitation.

### 3.8. Method Validation

The UHPLC-MS/MS method was fully validated according to the FDA guidelines on bioanalytical method validation [22]. The selectivity and specificity, carryover, linearity range, LOD, LOQ, intra-assay/inter-assay accuracy and precision, extraction recovery and matrix effect, dilution integrity, and stability under several conditions were determined.

#### 3.8.1. Selectivity, Specificity and Carry-Over

Selectivity was determined from the chromatograms of six individual blank plasma and homogenate matrices, compared to those of samples spiked with LLOQ concentration of analytes and IS, as well as the actual bio-samples after administration. The selectivity was considered to be good if the interference component in blank matrix samples accounted for no more than 20% of the analyte response and no more than 5% of the internal standard response in the respective LLOQ samples.

Specificity was demonstrated by analyzing the upper limit of quantification (ULOQ) in the samples without IS and zero calibrator samples (without analytes but with IS). Absence of interfering ingredients is accepted when the response is ≤20% of the analyte response and ≤5% of the IS response in the LLOQ sample.

Carry-over evaluation was performed by analyzing the double-blank sample (no analyte, no IS) after ULOQ concentration in plasma and tissue samples. In the case of a peak detected in the double-blank sample following the highest calibrator at the same retention time as the analyte and IS, the response of this peak should be no greater than 20% of the analyte and no greater than 5% of the IS in the LLOQ samples.

#### 3.8.2. Linearity, LOD and LOQ

To construct calibration curves, the ratio (y) between the peak area of analytes against internal standard versus the nominal analyte concentration (x) were plotted based on weighted least-squares. And the data was fitted via a weighted linear (1/x^2^) regression model, except for a quadratic (1/x^2^) regression model in the case of the albumin conjugate of legubicin in rat plasma. There should be a correlation coefficient (r) greater than 0.99. Signal-to-noise ratios of 3 and 10 were estimated by the analyst software for LOD and LOQ, respectively.

#### 3.8.3. Accuracy and Precision

The intra-batch and inter-batch accuracy and precision were assessed at LLOQ, LQC, MQC, and HQC levels in six biological replicates per QC level within each batch, and between different batches, respectively. The accuracy was determined through calculation of the deviation (bias) from the theoretical value and the precision was obtained through coefficient of variation (CV) among measured concentrations. The overall accuracy should not exceed a range of ±15% of the nominal concentration for each concentration level, except for LLOQ, which should not exceed a range of ±20.0%. The precision (%CV) should be within 15% for every concentration level, while for the LLOQ, it should be ≤20%.

#### 3.8.4. Extraction Recovery and Matrix Effect

Sets of six replicates at LQC, MQC, and HQC levels were used to evaluate the extraction recovery and matrix effect. Comparing the peak area ratio of each analyte-spiked blank matrix relative to its IS (R_pre-spiking_) with the ratios of standard solutions of equivalent concentrations spiked in post-extracted blank samples, relative to IS (R_post-spiking_), assessed recoveries, and these were calculated by the following equation: Extraction recovery (%) = R_pre-spiking_/R_post-spiking_ × 100%. A consistent and reproducible extraction recovery should be achieved regardless of concentration.

Analysis of matrix effects was performed by comparing the peak area ratios of analytes added to post-extracted blank matrices with those for analytes added to neat solutions (R_neat_) at equivalent concentrations of the analytes, according to the following equation: Matrix factor (%) = R_post-spiking_/R_neat_ × 100%. The matrix factor normalized by the internal standard was determined by calculating the ratio of the matrix factor of the analyte to the internal standard; its CV should be within 15%.

#### 3.8.5. Dilution Integrity

An investigation of the accuracy of biological matrix samples’ concentrations higher than the maximum calibration concentration was conducted using 10-fold and 20-fold dilutions of drug-free biological matrices. The average back-calculated concentration was required to fall within ±15% of the deviation from the actual value and its CV was not to exceed 15%.

#### 3.8.6. Stability Studies

Stability evaluations should cover the anticipated storage and analytical conditions. By investigating low- and high-concentration stability QCs in six replicates at different storage conditions, autosampler, short-term (bench-top), freeze and thaw, and long-term stability evaluations were conducted.

### 3.9. Animal Experiments

Twenty-four rats, inclusive of both sexes, were randomly distributed into four groups. Groups 1, 2, and 3 were single-dose groups, among which the subjects received legubicin via tail intravenous injection (a dose of 1.5, 5, or 15 mg/kg; i.e., 1.36, 4.52, or 13.6 µmol/kg). The fourth group was utilized for a multi-dose study at 5 mg/kg (once-weekly for 4 consecutive weeks). Blood samples of approximately 150 µL were taken from the jugular vein at 0 h (pre-dosing) and at 0.167 h (10 min), 0.5 h, 1 h, 2 h, 4 h, 8 h, 24 h, 48 h, and 72 h post-dosing in the single-dose groups. Samples were also obtained on days 1 and 22 for the multiple-dose group in the same way as for the single-dose groups. However, on days 8 and 15, samples were taken only before and 10 min after administration. The K2EDTA quantities containing blood samples were centrifuged (4 °C, 4000 rpm, 10 min) to collect plasma samples, which were separated into EP tubes and kept at −80 °C until further assay.

Seventy-two HT1080 tumor-bearing mice were randomly divided into 12 groups (*n* = 6), and half of the groups received legubicin and the other half received doxorubicin, both by tail vein injection, and both at a dose of 9.05 µmol/kg doxorubicin. Each legubicin administration group corresponded to a sampling time-point (4, 16, 32, 48, 96, 168, and 240 h after administration). Each doxorubicin treatment group was sampled 4, 16, 32, 48, and 96 h after dosing. Blood samples and tumor, heart, and kidney tissues were collected at the sampling time points (4, 16, 32, 48, 96, 168, and 240 h after dosing). In addition, liver, spleen, lung, stomach, small intestine, gonads, brain, skeletal muscle, and skin were also collected at 4, 32, 96, and 240 h post-dose time points in the legubicin administration group. All whole-blood samples were processed according to the same processing steps, described above, for rat whole-blood samples.

### 3.10. Data Analysis

The analysis software (Version 1.5.2, AB Sciex, Vaughan, ON, Canada) was applied to data acquisition and quantification. Pharmacokinetic parameters were calculated with WinNonlin Phoenix (v6.4, Pharsight, Mountain View, CA, USA) with the noncompartmental method, and were presented as mean ± SD (%CV). The “Best fit” strategy in the WinNonlin Phoenix was used for the calculation of elimination half-life and the selection of the elimination phase. Graphs were plotted by GraphPad Software (GraphPad Prism 8.0.2; GraphPad Software, Inc., La Jolla, CA, USA).

## 4. Conclusions

This study presents the establishment of a novel albumin-binding assay (enzyme digestion) and a simplified and efficient pretreatment method for free Leu-DOX and DOX. These methods offer a lower limit of quantification and reduced time requirements. The aforementioned UPLC-MS/MS method has been successfully validated and has demonstrated its reliability and reproducibility. This method effectively evaluated the plasma pharmacokinetics of legubicin in rats after intravenous administration and assessed tissue distribution in mice with tumors. Consequently, this study provides valuable insights for future investigations on legubicin.

## Figures and Tables

**Table 1:** Parameters for calibration curves of Leu-DOX, DOX, and albumin conjugate of legubicin in different matrices.

Matrix	Analyte	Calibration Range	Calibration Curve	r	LOD	LOQ
Rat plasma	Leu-DOX	5–5000 pmol/mL	y = 0.00432x + 0.00255	0.9978	0.261 pmol/mL	0.870 pmol/mL
	DOX	1.5–1500 pmol/mL	y = 0.01672x + 0.00190	0.9985	0.179 pmol/mL	0.595 pmol/mL
	Albumin conjugate of legubicin	0.1–100 nmol/mL	y = −0.00044x^2^ + 0.23550x + 0.00173	0.9979	0.006 nmol/mL	0.021 nmol/mL
Mouse plasma	Leu-DOX	3–3000 pmol/mL	y = 0.00007x − 0.00001	0.9975	0.667 pmol/mL	2.222 pmol/mL
	DOX	1–1000 pmol/mL	y = 0.00018x + 0.00003	0.9988	0.144 pmol/mL	0.481 pmol/mL
	Albumin conjugate of legubicin	0.1–100 nmol/mL	y = 0.00002x − 0.00004	0.9966	0.005 nmol/mL	0.018 nmol/mL
Mouse tumor	Leu-DOX	9–9000 pmol/mL	y = 0.00397x + 0.00561	0.9979	1.019 pmol/mL	3.396 pmol/mL
	DOX	3–3000 pmol/mL	y = 0.00313x + 0.00486	0.9971	0.709 pmol/mL	2.362 pmol/mL
Mouse tissue	Leu-DOX	9–9000 pmol/mL	y = 0.00348x + 0.00157	0.9928	2.000 pmol/mL	6.667 pmol/mL
	DOX	3–3000 pmol/mL	y = 0.00708x + 0.00016	0.9961	0.750 pmol/mL	2.500 pmol/mL

**Table 2:** Accuracy and precision of Leu-DOX, DOX, and albumin conjugate of legubicin in rat plasma (6 replicates per day for three days).

Analytes	Nominal Concentration ^a^	Intra-Day									Inter-Day		
		1st Day			2nd Day			3rd Day					
		Measured Concentration (Mean ± SD) ^a^	Accuracy (% Bias)	Precision (% RSD)	Measured Concentration (Mean ± SD) ^a^	Accuracy (% Bias)	Precision (% RSD)	Measured Concentration (Mean ± SD) ^a^	Accuracy (% bias)	Precision (% RSD)	Measured Concentration ^a^	Accuracy (% bias)	Precision (% RSD)
Leu-DOX	5	5.61 ± 0.30	12.2	5.4	5.55 ± 0.35	11.0	6.4	5.03 ± 0.31	0.5	6.2	5.40 ± 0.41	7.9	7.5
	10	9.52 ± 0.53	−4.8	5.6	9.86 ± 0.66	−1.4	6.7	10.26 ± 0.59	2.6	5.8	9.88 ± 0.64	−1.2	6.5
	200	183.00 ± 13.97	−8.5	7.6	193.17 ± 8.45	−3.4	4.4	189.50 ± 8.89	−5.3	4.7	188.56 ± 10.97	−5.7	5.8
	4000	3688.33 ± 210.09	−7.8	5.7	3876.67 ± 122.26	−3.1	3.2	3930.00 ± 309.13	−1.8	7.9	3831.67 ± 238.46	−4.2	6.2
DOX	1.5	1.49 ± 0.07	−0.5	4.5	1.41 ± 0.05	−6.3	3.7	1.44 ± 0.10	−4.2	6.8	1.44 ± 0.08	−3.7	5.5
	3	2.91 ± 0.10	−3.0	3.4	2.90 ± 0.13	−3.3	4.6	2.84 ± 0.12	−5.5	4.2	2.88 ± 0.12	−3.9	4.0
	60	54.73 ± 4.06	−8.8	7.4	55.03 ± 4.30	−8.3	7.8	58.65 ± 1.12	−2.3	1.9	56.14 ± 3.74	−6.4	6.7
	1200	1106.67 ± 58.54	−7.8	5.3	1143.33 ± 75.28	−4.7	6.6	1138.33 ± 31.89	−5.1	2.8	1129.44 ± 57.03	−5.9	5.0
Albumin conjugate of legubicin	0.1	0.11 ± 0.01	8.0	5.6	0.10 ± 0.01	1.0	7.9	0.11 ± 0.00	5.8	6.3	0.11 ± 0.01	5.8	6.3
	0.2	0.21 ± 0.01	6.5	4.2	0.20 ± 0.01	1.5	4.9	0.21 ± 0.01	4.2	4.8	0.21 ± 0.01	4.2	4.8
	4	4.39 ± 0.39	9.7	9.0	4.11 ± 0.26	2.7	6.4	4.25 ± 0.36	6.3	8.0	4.25 ± 0.34	6.3	8.0
	80	91.07 ± 6.55	13.8	7.2	91.77 ± 3.79	14.7	4.1	80.73 ± 5.29	9.8	8.2	87.86 ± 7.21	9.8	8.2

^a^ pmol/mL for Leu-DOX/DOX, nmol/mL for albumin conjugate of legubicin.

**Table 3:** The extraction recovery and matrix effect of analytes in rat plasma (*n* = 6 for each concentration).

Analytes	Nominal Concentration ^a^	Extraction Recovery (%, Mean ± SD)	RSD (%)	Matrix Factor (%, Mean ± SD)	RSD (%)
Leu-DOX	10	102.1 ± 6.5	6.4	99.4 ± 10.8	10.9
	200	98.2 ± 3.2	3.3	98.7 ± 7.0	7.0
	4000	96.6 ± 5.4	5.6	96.5 ± 5.5	5.7
DOX	3	93.7 ± 8.4	8.9	108.8 ± 4.5	4.1
	60	97.8 ± 3.2	3.3	98.6 ± 2.7	2.8
	1200	96.9 ± 7.9	8.2	103.6 ± 9.6	9.3
Albumin conjugate of legubicin	0.2	94.1 ± 4.5	4.8	84.3 ± 3.2	3.8
	4	90.0 ± 6.1	6.8	99.4 ± 7.6	7.7
	80	89.7 ± 8.8	9.9	107.4 ± 5.9	5.5

^a^ pmol/mL for Leu-DOX/DOX, nmol/mL for albumin conjugate of legubicin.

**Table 4:** Stability of Leu-DOX, DOX, and albumin conjugate of legubicin, under different storage conditions, in rat plasma (*n* = 6).

Analytes	Nominal Concentration ^a^	Short-Term Stability at Room Temperature (4 h)			Freeze and Thaw Stability at −80 °C/Room Temperature (3 Cycles)			Auto-Sampler Stability at 2–8 °C (72 h)			Long-Term Stability at −80 °C (70 days)		
		Measured Concentration (Mean ± SD) ^a^	Bias (%)	RSD (%)	Measured Concentration (Mean ± SD) ^a^	Bias (%)	RSD (%)	Measured Concentration (Mean ± SD) ^a^	Bias (%)	RSD (%)	Measured Concentration (Mean ± SD) ^a^	Bias (%)	RSD (%)
Leu-DOX	10	9.76 ± 0.41	−2.4	4.2	10.20 ± 0.70	2.0	6.8	9.96 ± 0.48	−0.4	4.8	10.07 ± 1.50	0.7	15.0
	4000	3871.67 ± 390.25	−3.2	10.1	3775.00 ± 329.89	−5.6	8.7	3758.33 ± 422.58	−6.0	11.2	4300.00 ± 240.83	7.5	5.6
DOX	3	3.04 ± 0.18	1.2	5.9	3.01 ± 0.15	0.4	5.1	3.13 ± 0.25	4.4	7.9	2.98 ± 0.29	−0.6	9.7
	1200	1238.33 ± 100.48	3.2	8.1	1221.67 ± 97.45	1.8	8.0	1211.67 ± 104.58	1.0	8.6	1190.00 ± 40.50	−0.8	3.4
Albumin conjugate of legubicin	0.2	0.19 ± 0.01	−7.1	5.8	0.18 ± 0.01	−10.6	5.3	0.18 ± 0.01	−8.7	4.2	0.17 ± 0.00	−13.7	2.2
	80	87.30 ± 8.98	9.1	10.3	81.23 ± 2.80	1.5	3.5	80.17 ± 2.65	0.2	3.3	84.62 ± 9.77	5.8	11.5

^a^ pmol/mL for Leu-DOX/DOX, nmol/mL for albumin conjugate of legubicin.

**Table 5:** The main pharmacokinetic parameters after intravenous administration of a single dose of legubicin (*n* = 6 per group, mean ± SD), ^$^: presented as median [min, max].

Albumin conjugate of legubicin PK parameters	1.5 mg/kg	5 mg/kg	15 mg/kg			
AUC_INF_obs_ (h*pmol/mL)	222,000 ± 47,600	873,000 ± 155,000	2,020,000 ± 331,000			
AUC_last_ (h*pmol/mL)	218,000 ± 47,400	869,000 ± 152,000	2,000,000 ± 324,000			
C_0_ (pmol/mL)	30,900 ± 7290	96,300 ± 19,300	224,000 ± 48,100			
Cl__obs_ (mL/h/kg)	6.4 ± 1.53	5.33 ± 1.05	6.87 ± 1.16			
C_max_ (pmol/mL)	28,900 ± 6310	97,300 ± 8270	204,000 ± 39,800			
t_1/2z_ (h)	8.43 ± 2.6	9.72 ± 1.01	10.7 ± 2.31			
T_max_ $ (h)	0.167 [0.167, 0.167]	0.334 [0.167, 1]	0.167 [0.167, 0.167]			
V_ss_obs_ (mL/kg)	66.7 ± 9.85	59.2 ± 5.7	98.6 ± 19.3			
V_z_obs_ (mL/kg)	73.5 ± 10.4	73.8 ± 9.09	105 ± 26.2			
Leu-DOX PK parameters	1.5 mg/kg, Female	1.5 mg/kg, Male	5 mg/kg, Female	5 mg/kg, Male	15 mg/kg, Female	15 mg/kg, Male
AUC_last_ (h*pmol/mL)	6.44 ± 2.02	11.2 ± 2.1	52.0 ± 16.2	94.3 ± 10.5	336 ± 62.5	1030 ± 244
C_max_ (pmol/mL)	21.5 ± 7.78	29.5 ± 2.74	72.1 ± 16.0	145 ± 23.9	373 ± 63.2	1100 ± 243
T_max_ $ (h)	0.167 [0.167, 0.167]	0.167 [0.167, 0.167]	0.167 [0.167, 0.167]	0.167 [0.167, 0.167]	0.167 [0.167, 0.167]	0.167 [0.167, 0.167]
DOX PK parameters	5 mg/kg, Female	5 mg/kg, Male	15 mg/kg, Female	15 mg/kg, Male		
AUC_last_ (h*pmol/mL)	100 ± 5.38	173 ± 24.3	486 ± 46.4	789 ± 99.7		
C_max_ (pmol/mL)	3.11 ± 0.615	6.65 ± 1.30	20.5 ± 5.06	44.2 ± 4.65		
T_max_ $ (h)	0.167 [0.167, 0.167]	0.167 [0.167, 0.167]	0.167 [0.167, 0.167]	0.167 [0.167, 0.167]		

**Table 6:** The pharmacokinetic parameters of single dose (day 1) treatments versus multiple dose treatments (day 22) following 4 consecutive weeks of weekly administration (5 mg/kg) (*n* = 6 in each group), ^$^: presented as median [min, max].

Albumin conjugate of legubicin PK parameters	Single dose	Multiple dose		
AUC_INF_obs_ (h*pmol/mL)	563,000 ± 91,200	814,000 ± 156,000		
AUC_last_ (h*pmol/mL)	560,000 ± 90,900	808,000 ± 156,000		
C_0_ (pmol/mL)	75,600 ± 17,600	80,400 ± 20,500		
Cl__obs_ (mL/h/kg)	8.21 ± 1.35	5.75 ± 1.23		
C_max_ (pmol/mL)	68,700 ± 15,600	88,600 ± 12,200		
t_1/2z_ (h)	9.32 ± 0.994	10.0 ± 1.63		
T_max_^$^ (h)	0.167 [0.167, 0.167]	0.5 [0.167, 1]		
V_ss_obs_ (mL/kg)	97.7 ± 23.2	67.6 ± 9.15		
V_z_obs_ (mL/kg)	110 ± 18.3	81.5 ± 12.8		
Leu-DOX PK parameters	Single dose, female	Single dose, male	Multiple dose, female	Multiple dose, male
AUC_last_ (h*pmol/mL)	38.7 ± 1.04	81.0 ± 13.3	60.4 ± 6.43	112 ± 14.0
C_max_ (pmol/mL)	60.0 ± 6.89	111 ± 13.5	80.3 ± 8.57	141 ± 22.0
T_max_^$^ (h)	0.167 [0.167, 0.167]	0.167 [0.167, 0.167]	0.167 [0.167, 0.167]	0.167 [0.167, 0.167]
DOX PK parameters	Single dose, female	Single dose, male	Multiple dose, female	Multiple dose, male
AUC_last_ (h*pmol/mL)	94.5 ± 44.7	123 ± 44.1	174 ± 36.7	207 ± 11.2
C_max_ (pmol/mL)	3.10 ± 0.367	6.01 ± 0.430	4.70 ± 0.534	8.58 ± 0.616
T_max_^$^ (h)	0.167 [0.167, 0.167]	0.167 [0.167, 0.167]	0.167 [0.167, 0.167]	0.167 [0.167, 0.167]

**Table 7:** A summary of parameters of doxorubicin production in heart, kidney, tumor, and plasma, after intravenous administration of legubicin or doxorubicin, in tumor-bearing mice (*n* = 6).

	Legubicin Administration		Doxorubicin Administration	
Plasma/Tissue	AUC_last_ (h*nmol/g)	T/P Ratio	AUC_last_ (h*nmol/g)	T/P Ratio
Plasma	0.835	1.0	1.07	1.0
Heart	8.89	10.7	29.0	27.1
Tumor	112	134.1	63.1	59.0
Kidney	24.1	28.9	83.5	78.0

**Table 8:** Mass parameters for Leu-DOX, DOX, and phenacetin.

Biological Matrix	Analyte	Q1 ^a^ (*m*/*z*)	Q3 ^b^ (*m*/*z*)	DP ^c^ (V)	CE ^d^ (V)	CXP ^e^ (V)	EP ^f^ (V)	Dwell Time (ms)
Rat plasma	Leu-DOX	657.5	243.1	70	18	13	10	100
	DOX	544.0	397.0	98	18	13	10	100
	Phenacetin	180.0	110.1	120	27	13	10	100
Mouse plasma/tumor/tissue	Leu-DOX	657.4	243.2	70	25	13	10	50
	DOX	544.2	397.2	100	16	13	10	50
	Phenacetin	180.1	110	90	27	13	10	50

^a^ Q1, precursor ion. ^b^ Q3, product ion. ^c^ DP, de-clustering potential. ^d^ CE, collision energy. ^e^ CXP, cell exit potential. ^f^ EP, entrance potential.

## Data Availability

The data are contained within the article and Supplementary Materials.

## Supplementary Materials

The following supporting information can be downloaded at: https://www.mdpi.com/article/10.3390/molecules29040775/s1, Figure S1: For free Leu-DOX and DOX: Typical MRM chromatograms of (a) rat blank plasma, (b) plasma sample containing LLOQ of Leu-DOX (5 pmol/mL) and DOX (1.5 pmol/mL) with IS added, and (c) plasma sample of rat taken at 30 min following administration of a single dose of legubicin at 1.5 mg/kg, with IS added. For total Leu-DOX after enzymatic hydrolysis by Legumain: Typical MRM chromatograms of (d) rat blank plasma, (e) legubicin-spiked plasma sample at LLOQ (0.1 nmol/mL) with IS added, and (f) plasma sample of rat taken at 30 min following administration of a single dose of legubicin at 1.5 mg/kg, with IS added; Figure S2: Product ion mass spectra of Leu-DOX (a), DOX (b) and IS (c); Table S1: Accuracy and precision of analytes in mouse biomatrices (6 replicates per concentration); Table S2: The extraction recovery and matrix effect of analytes in mouse biomatrices (*n* = 6 for each concentration); Table S3: Dilution integrity of analytes in rat and mouse biomatrices (*n* = 6); Table S4: Stability of analytes under different storage conditions in mouse biomatrices (*n* = 6); Table S5: Results of 90% confidence interval of exposure parameters (AUC) of Legubicin on dose linear regression (Power model) parameters.
