# Supplementary figures and images for: UHPLC-MS/MS Assay for Quantification of Legubicin, a Novel Doxorubicin-Based Legumain-Activated Prodrug, and Its Application to Pharmacokinetic and Tissue Distribution Studies
Source: Molecules. 2024 Feb 8;29(4):775. doi: 10.3390/molecules29040775 (PMC10892419; doi:10.3390/molecules29040775)

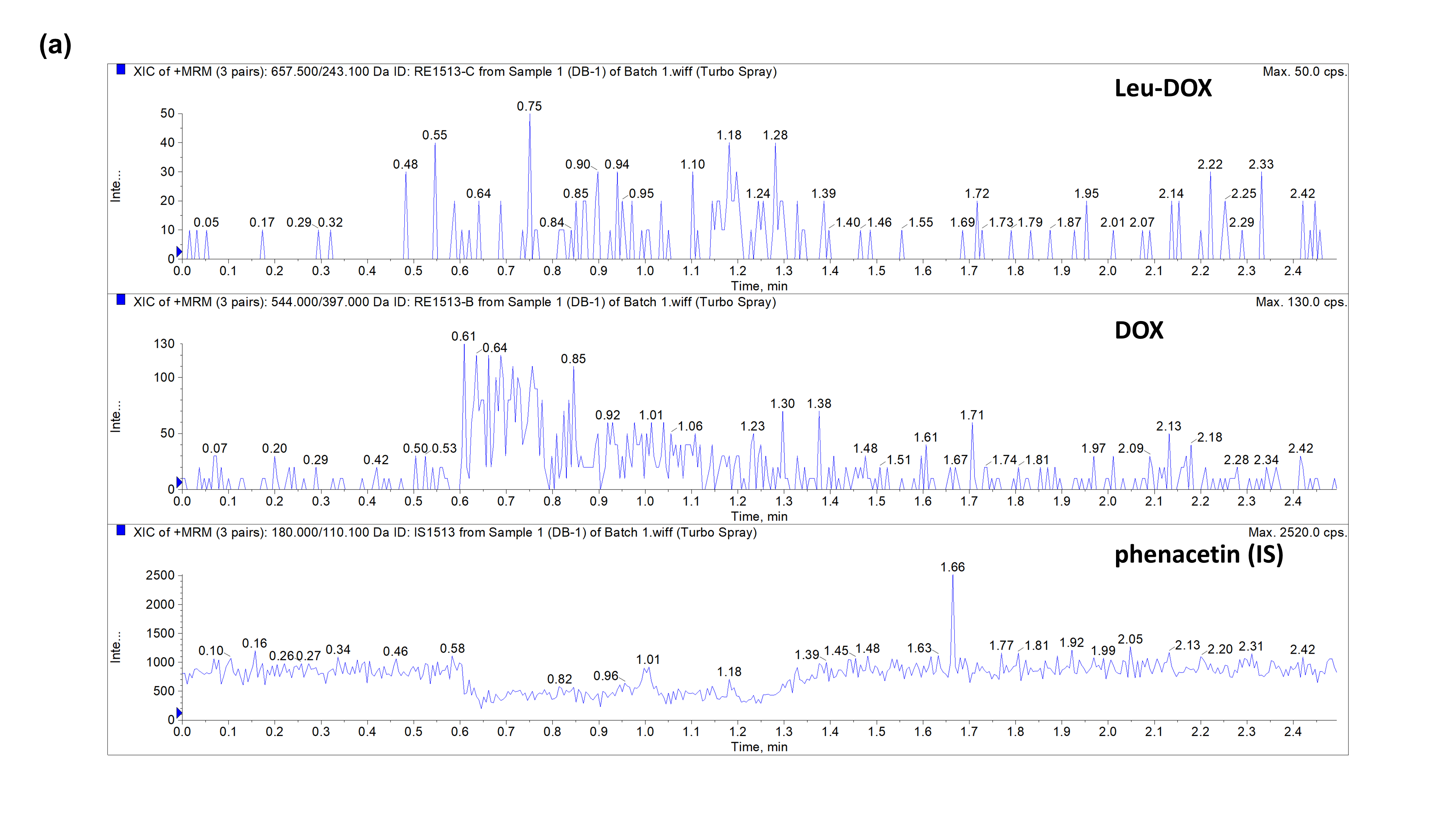

Supplement: Supplementary file 1 [file molecules-29-00775-s001.zip › Supplementary figures/Figure S1. a.TIF]

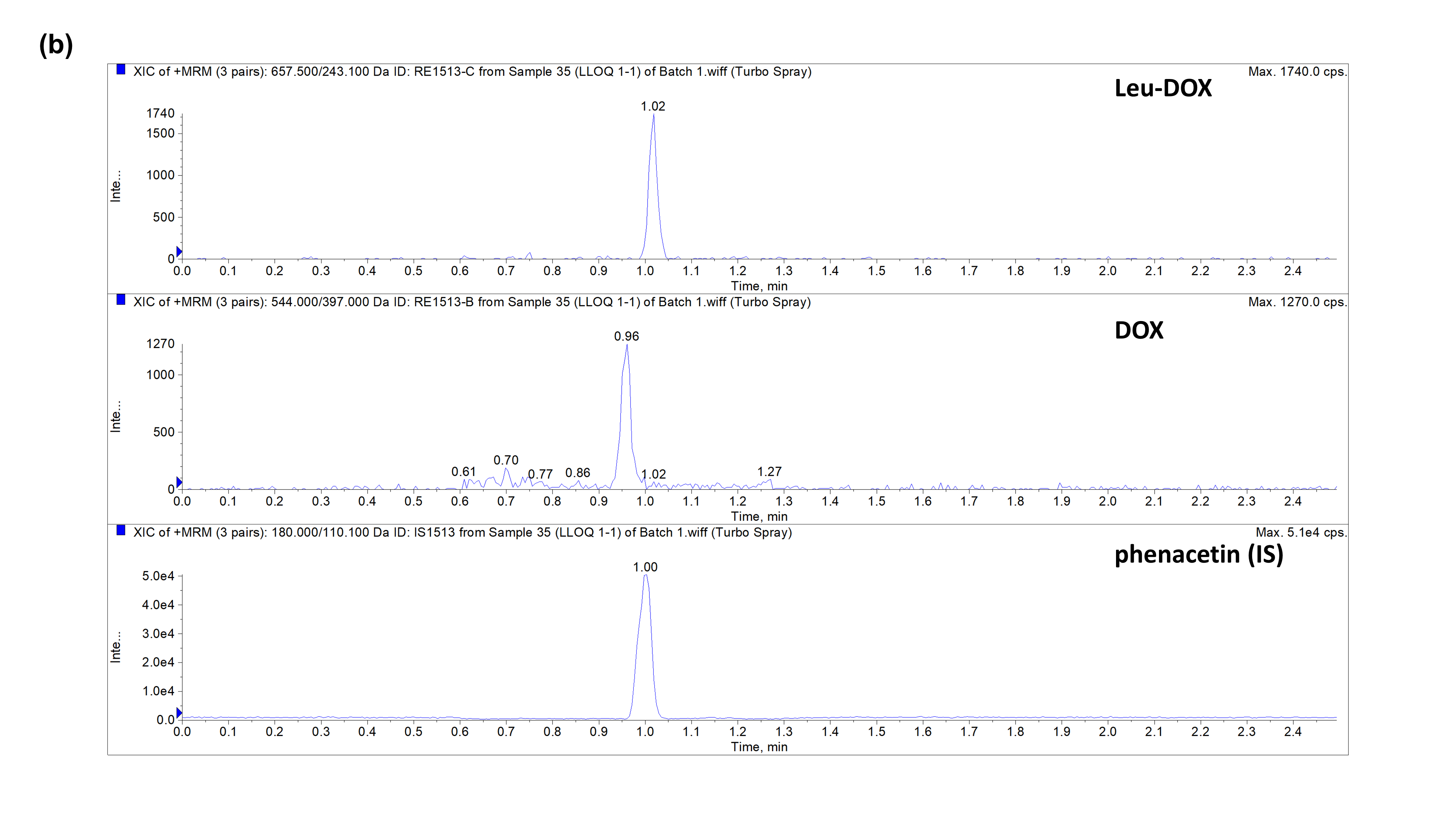

Supplement: Supplementary file 1 [file molecules-29-00775-s001.zip › Supplementary figures/Figure S1. b.TIF]

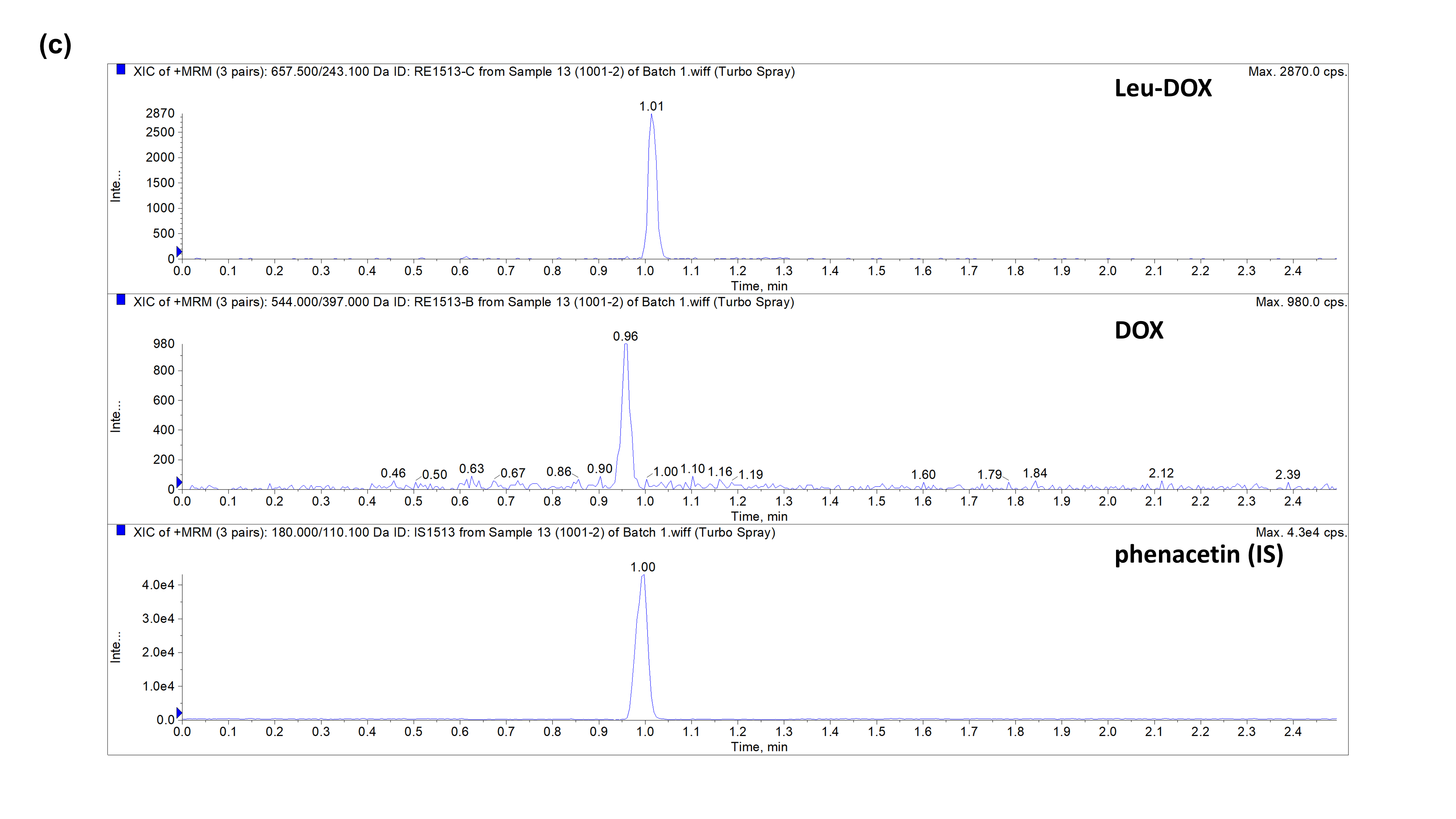

Supplement: Supplementary file 1 [file molecules-29-00775-s001.zip › Supplementary figures/Figure S1. c.TIF]

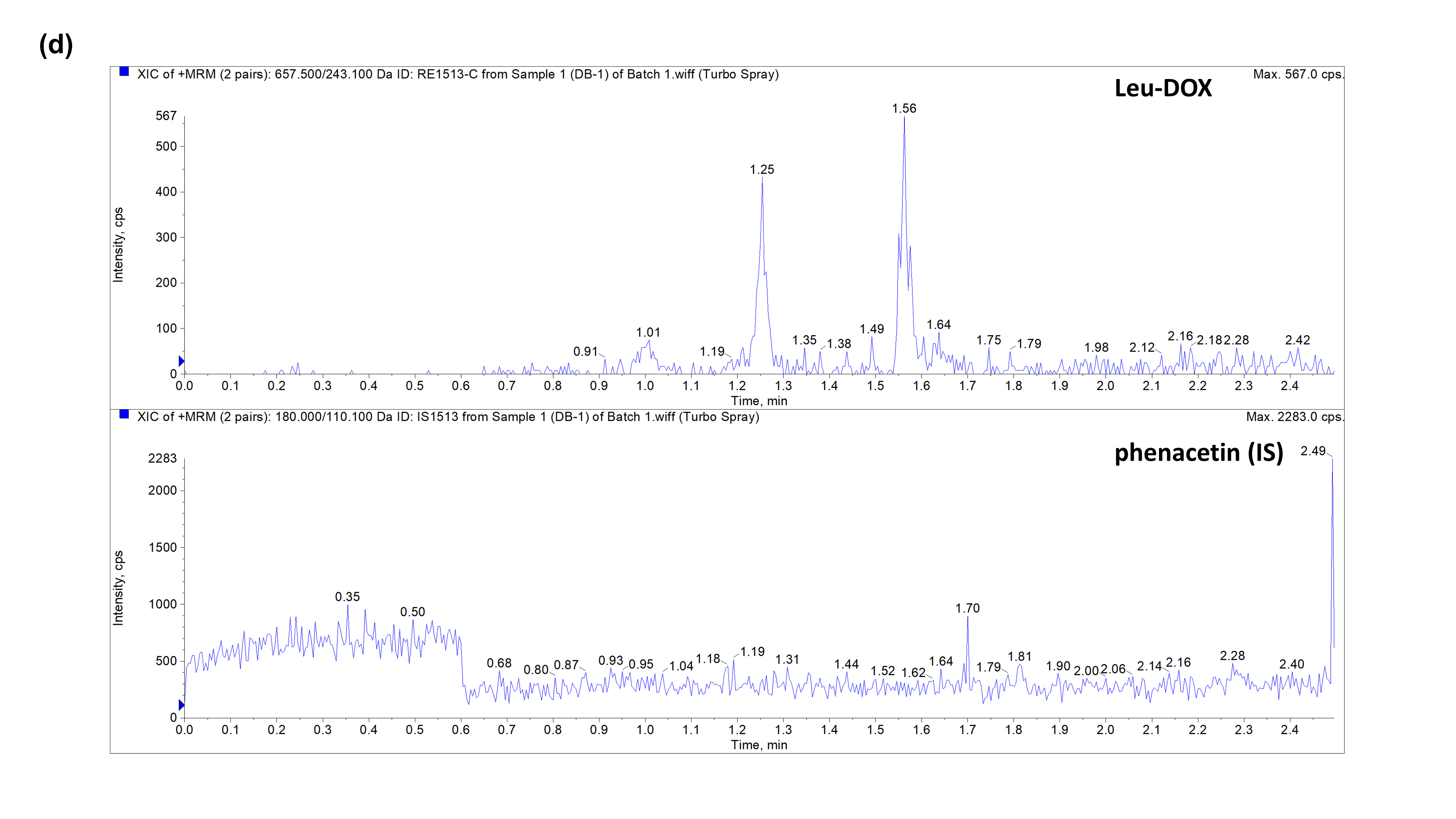

Supplement: Supplementary file 1 [file molecules-29-00775-s001.zip › Supplementary figures/Figure S1. d.TIF]

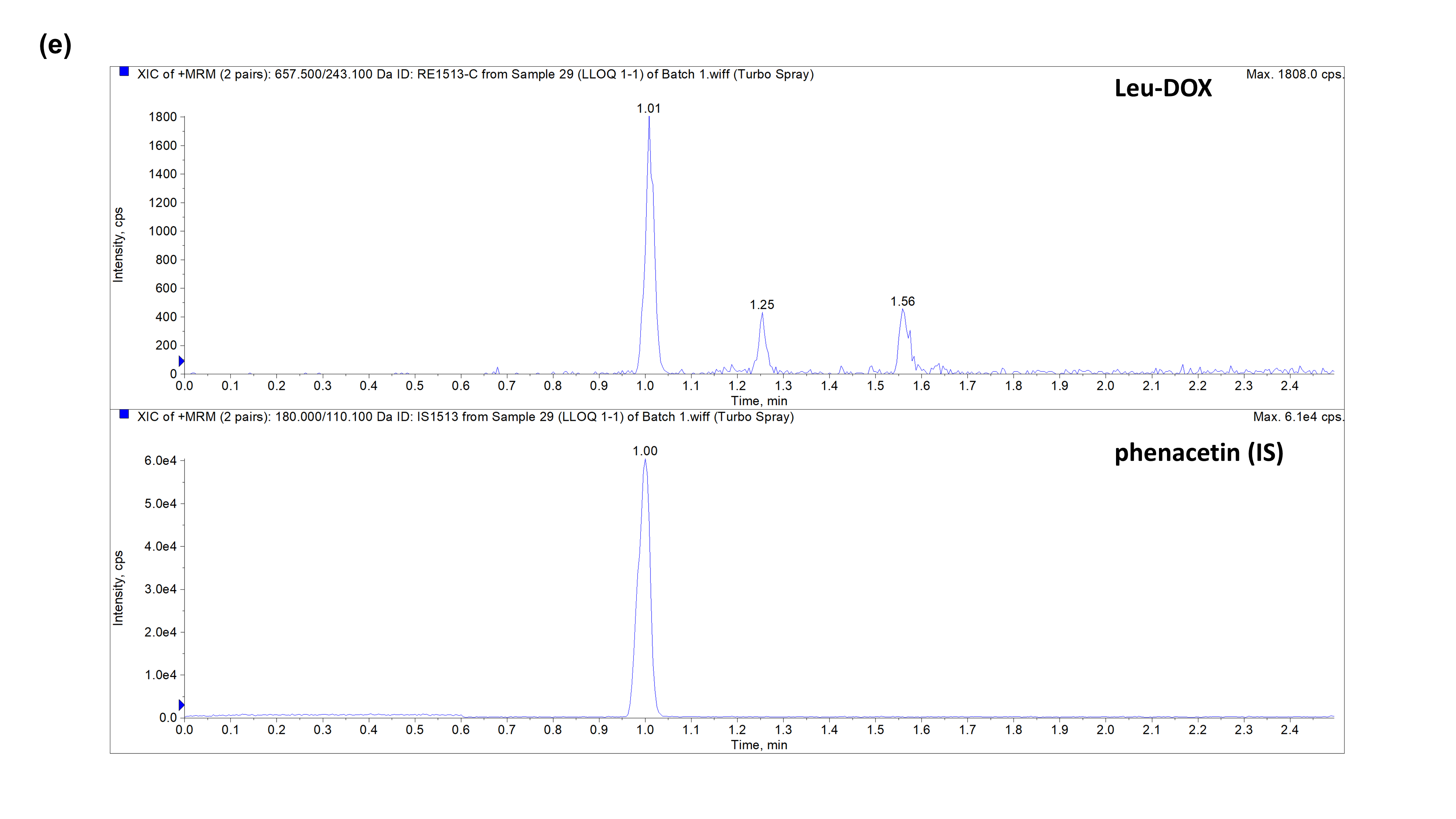

Supplement: Supplementary file 1 [file molecules-29-00775-s001.zip › Supplementary figures/Figure S1. e.TIF]

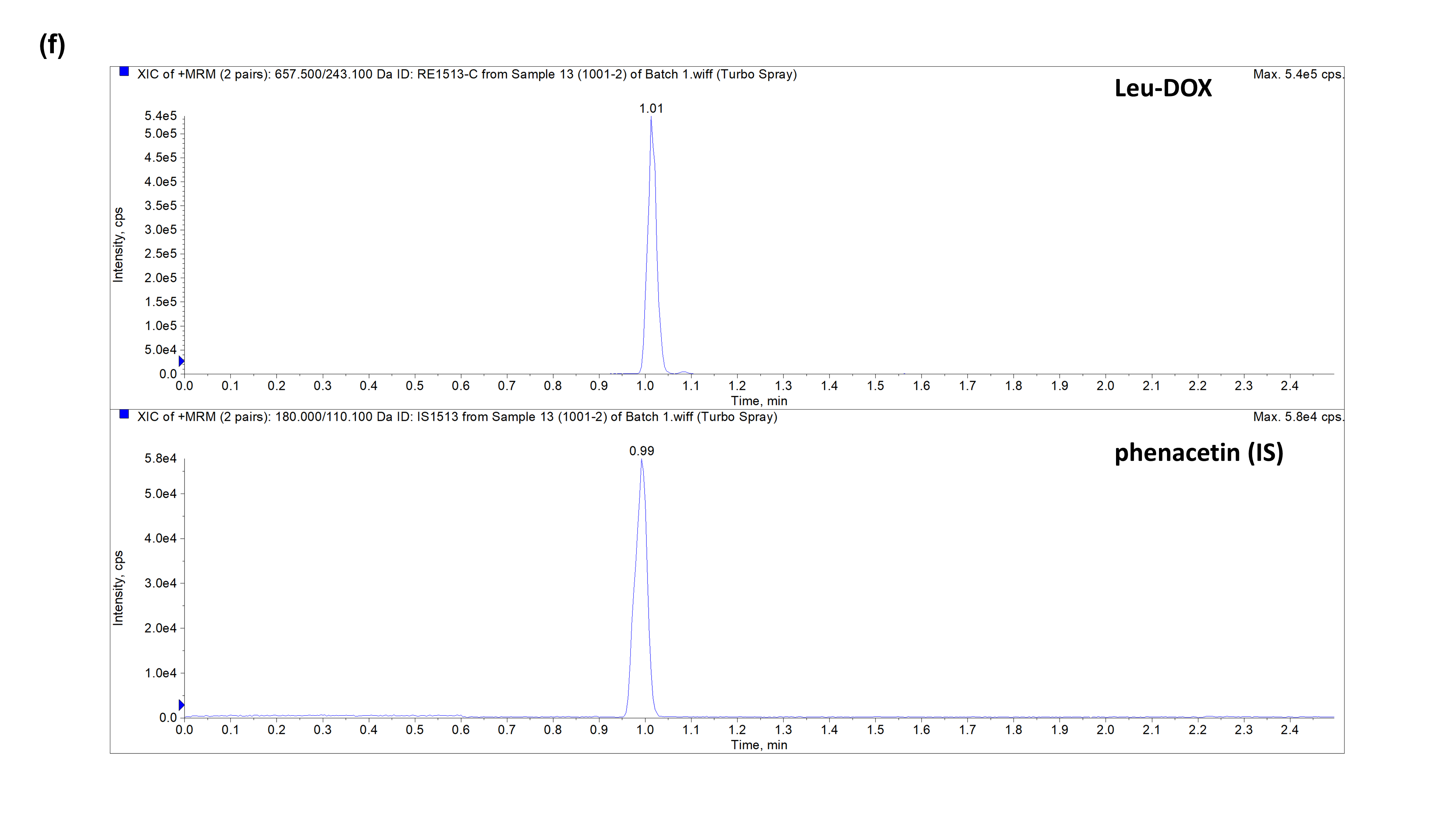

Supplement: Supplementary file 1 [file molecules-29-00775-s001.zip › Supplementary figures/Figure S1. f.TIF]

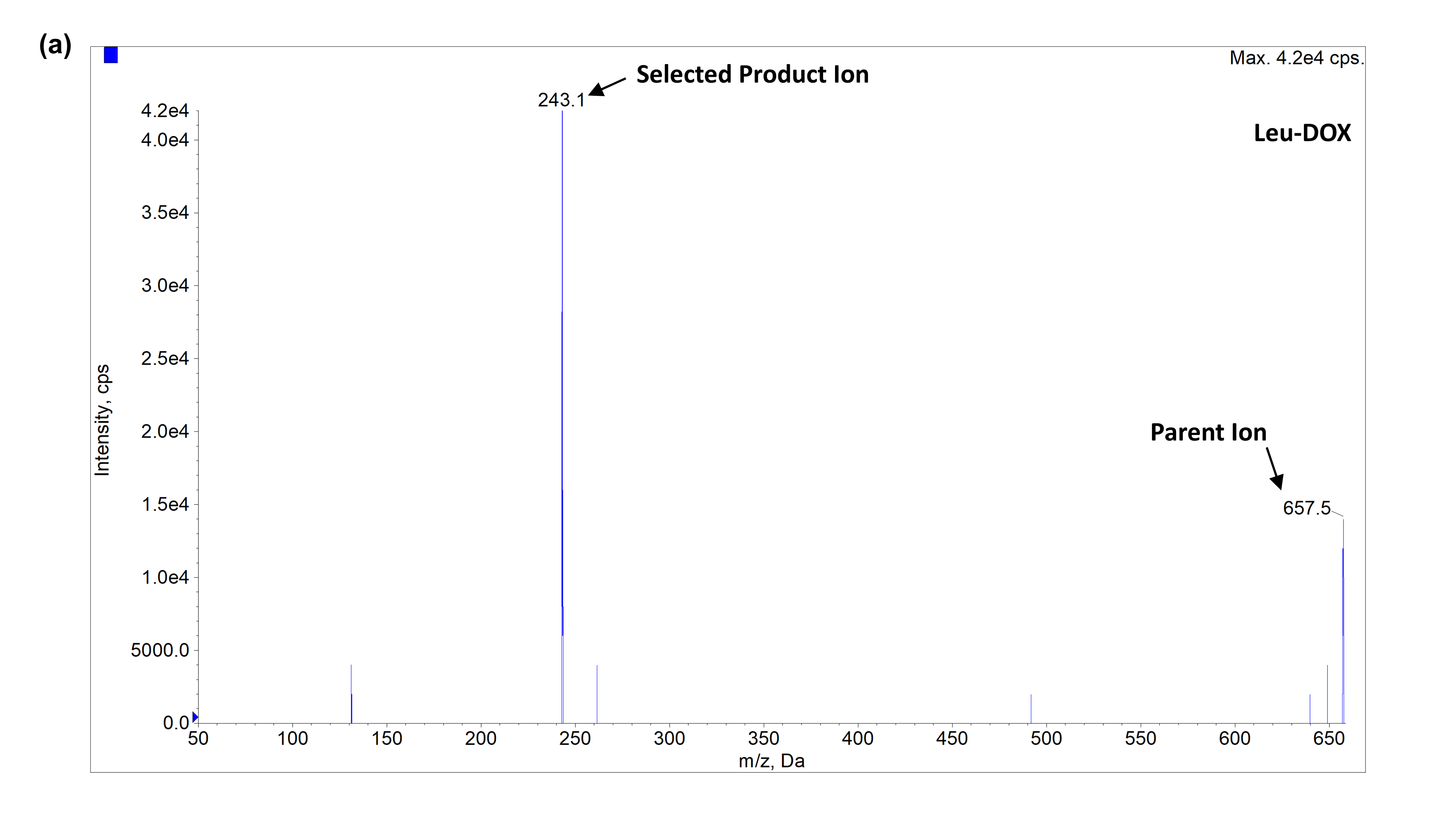

Supplement: Supplementary file 1 [file molecules-29-00775-s001.zip › Supplementary figures/Figure S2. a.TIF]

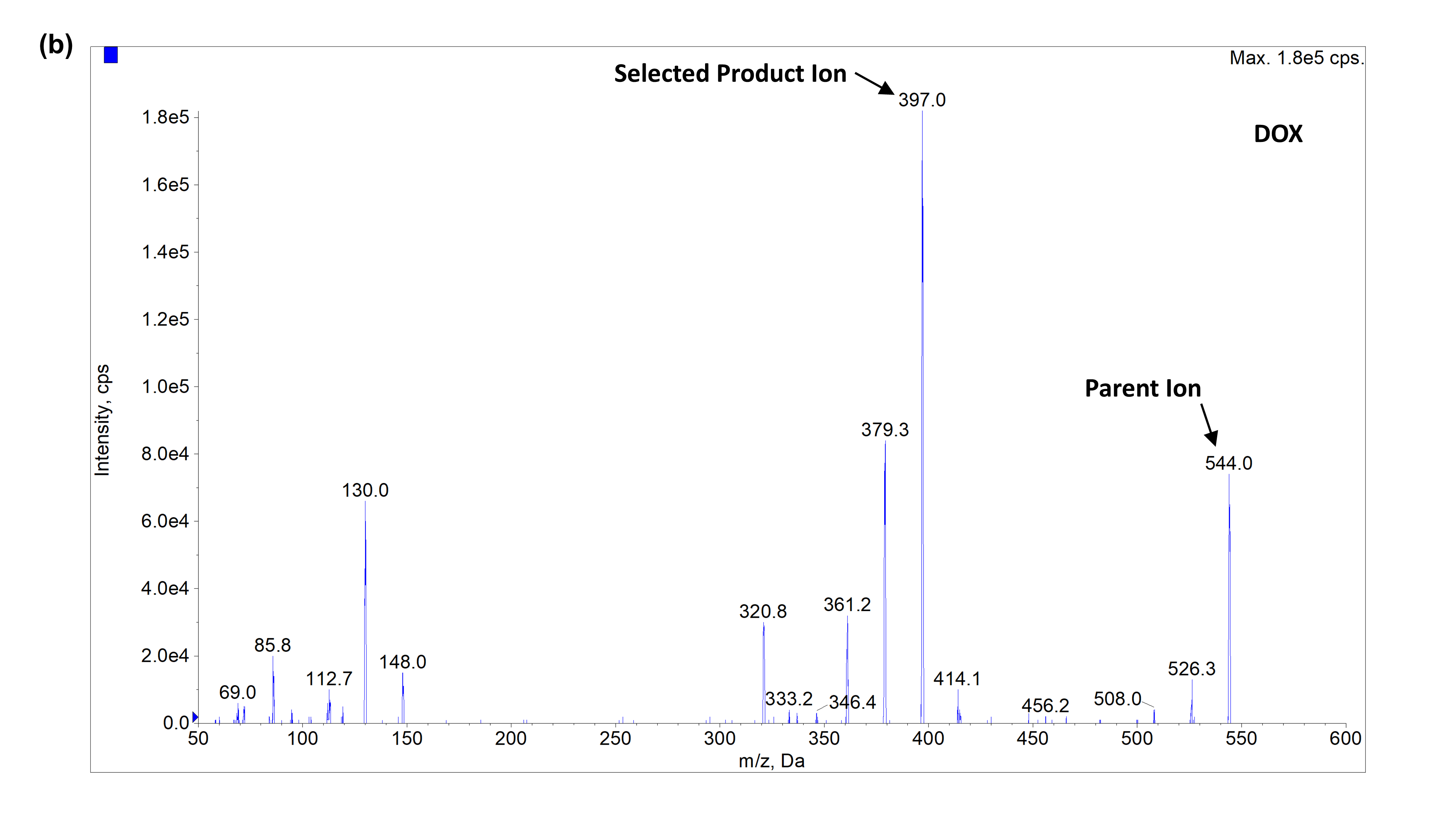

Supplement: Supplementary file 1 [file molecules-29-00775-s001.zip › Supplementary figures/Figure S2. b.TIF]

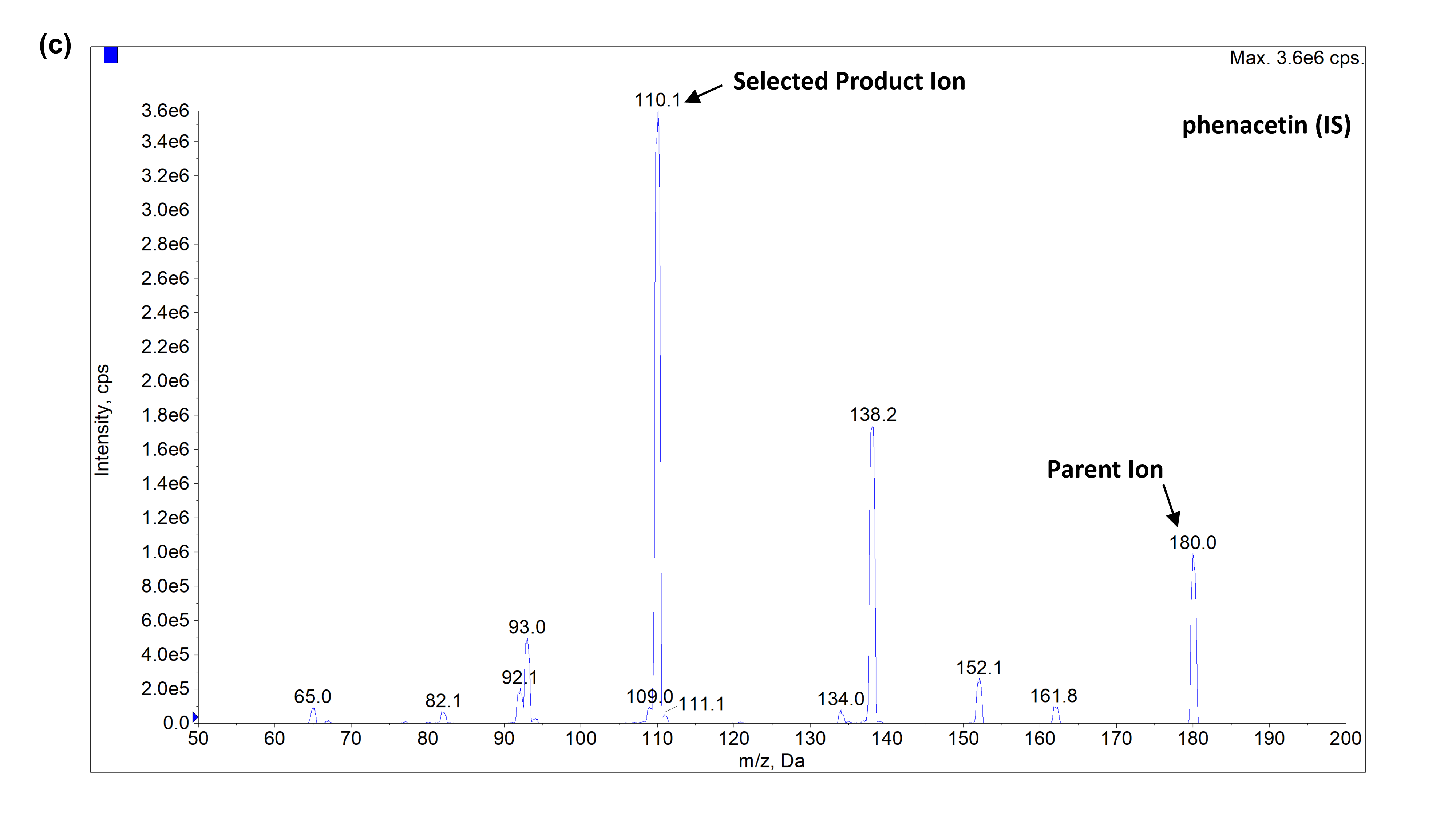

Supplement: Supplementary file 1 [file molecules-29-00775-s001.zip › Supplementary figures/Figure S2. c.TIF]
